# Sequence-selective encapsulation and protection of long peptides by a self-assembled Fe^II^_8_L_6_ cubic cage

**DOI:** 10.1038/ncomms14882

**Published:** 2017-03-30

**Authors:** Jesús Mosquera, Bartosz Szyszko, Sarah K. Y. Ho, Jonathan R. Nitschke

**Affiliations:** 1 Department of Chemistry, University of Cambridge, Lensfield Road, Cambridge CB2 1EW, UK

## Abstract

Self-assembly offers a general strategy for the preparation of large, hollow high-symmetry structures. Although biological capsules, such as virus capsids, are capable of selectively recognizing complex cargoes, synthetic encapsulants have lacked the capability to specifically bind large and complex biomolecules. Here we describe a cubic host obtained from the self-assembly of Fe^II^ and a zinc-porphyrin-containing ligand. This cubic cage is flexible and compatible with aqueous media. Its selectivity of encapsulation is driven by the coordination of guest functional groups to the zinc porphyrins. This new host thus specifically encapsulates guests incorporating imidazole and thiazole moieties, including drugs and peptides. Once encapsulated, the reactivity of a peptide is dramatically altered: encapsulated peptides are protected from trypsin hydrolysis, whereas physicochemically similar peptides that do not bind are cleaved.

## Abstract (web summary)

One of the challenges of synthetic self-assembled capsules is achieving selective recognition of specific cargoes. Here, authors synthesize a self-assembled porphyrin cubic cage that is capable of sequestering imidazole and thiazole-containing small molecules and peptides, protecting them from proteolysis.

Much recent effort has been directed towards the exploration of new functions enabled by synthetic encapsulants^1^,^2^. Confinement can stabilize reactive species^3^, promote unusual reactivity^4^, modulate catalyst activity^5^,^6^ or report on the presence of an analyte in a sensor^7^. These functions have been explored mainly using guests smaller than 1 nm in length^8^, largely because of the difficulties related to the preparation of large host molecules, which are capable of effectively enclosing a volume that experiences an environment distinct from the surrounding solvent. With recent refinements in the techniques of molecular self-assembly, this limitation is being overcome^9^,^10^.

The largest synthetic self-assembled structures that have been reported were not observed to serve as encapsulants for guest molecules^11^,^12^,^13^,^14^. This lack of host–guest binding might be understood in terms of: the rigidity of the hosts preventing adaptation that would maximize stabilizing contact with the guests; a low degree of cavity enclosure, which results in a low number of interactions between guest and host; and the absence of complementary moieties within the walls of the host to bind guests through specific interactions. The direct binding of large, information-rich biomolecules, such as natural biopolymers, has thus not been achieved^8^, despite the applications that such binding could enable. In particular, of the many therapeutic peptides and proteins that have been discovered, only a few are in current use due to their low bioavailability and susceptibility to enzymatic degradation^15^,^16^. Synthetic self-assembled capsules could mitigate these limitations by altering the solubility of peptides and protecting them from cleavage, following the example of previous successful interfaces between abiological and biological systems^17^,^18^. However, the only examples of the encapsulation of such molecules reported to date involve the covalent, irreversible attachment of a protein to the inside of a cage, preventing release or exchange^19^, or the encapsulation of short peptides^20^,^21^.

Here we report the preparation and characterization of the large, porphyrin-faced cubic cage **1**. Its flexibility and the incorporation of porphyrinatozinc(II) centres enable the tight, specific and reversible binding of guests incorporating imidazole motifs, including histidine-containing peptides up to 23 residues in length. Following encapsulation, guest peptides were protected from trypsin cleavage, whereas non-encapsulated peptides present in the same solution were rapidly cleaved. Cage **1** thus represents the first example of an artificial host able to bind large and complex biomolecular guests, altering their behaviour in fundamental ways.

## Results

### Synthesis and characterization of 1

**Figure 1:**
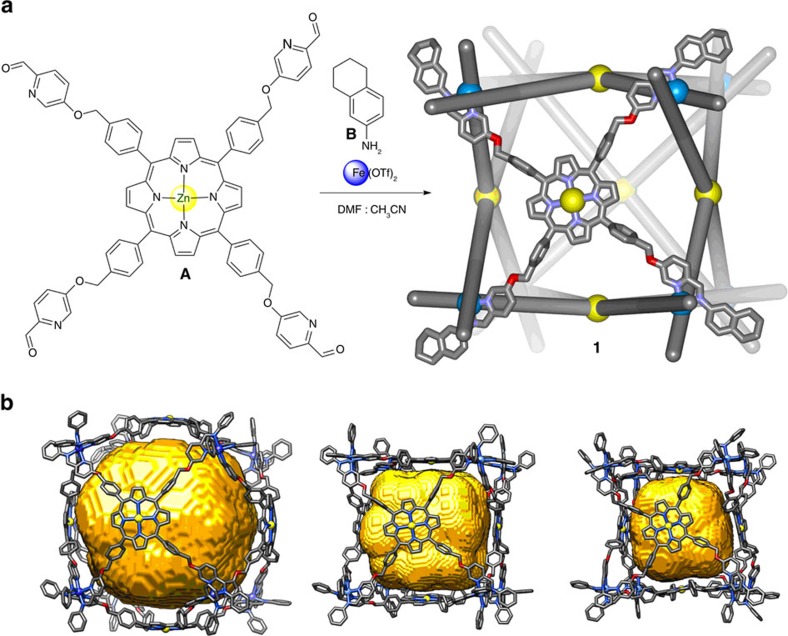
Synthesis and views of 1. (**a**) The one-pot synthetic procedure leading from 6 equiv. of porphyrin **A**, 24 equiv. of tetrahydronaphthylamine **B** and 8 equiv. of iron(II) trifluoromethanesulfonate to cube **1**. Only one face of the cube is shown in ball-and-stick form for clarity. (**b**) Ball-and-stick representations of three different MM2 energy-minimized models of **1** (not showing hydrogen atoms), having void volumes (shown in yellow) of 10,083, 5,600 and 3,143 Å^3^, respectively, the range of which is attributed to the flexibility of **1**. The fused cyclic parts of the aniline residues are omitted for clarity in (**b**). DMF, dimethylformamide; OTf, trifluoromethanesulfonate.

Capsule **1** was the uniquely observed product following the reaction between tetrakis(formylpyridine)porphyrin **A** (6 equiv.), tetrahydronaphthylamine **B** (24 equiv.) and iron(II) trifluoromethanesulfonate (8 equiv.), in a 4:1 mixture of DMF and MeCN (Fig. 1a). The ^1^H NMR spectrum of **1** was consistent with an *O*-symmetrical face-capped cubic structure (Supplementary Fig. 5). Confirmation of the Fe^II^_8_L_6_ formulation for **1** was provided by electrospray ionization mass spectrometry (Supplementary Figs 11 and 12).

Efforts to grow X-ray quality crystals of **1** were unsuccessful, possibly owing to its flexibility. Therefore, to gain insight into the three-dimensional structure of **1** we prepared an MM2 energy-minimized model (Fig. 1b). By starting with configurations in which the porphyrins were spaced closer or farther from the centre, energy-minimized models provided estimations of this volume that ranged between 3,000 and 10,000 Å^3^ (Supplementary Figs 57–59 and Supplementary Methods), with Fe^II^···Fe^II^ distances along the cube edges ranging from 20–22 Å. Diffusion-ordered spectroscopy nuclear magnetic resonance (DOSY NMR) analysis provided a hydrodynamic radius of 19.7 Å, within the range of 17–21 Å derived from the models (Supplementary Figs 10 and 57–59). The internal volume available for guest binding is thus anticipated to depend strongly on the solution conformation adopted. Cage **1** thus combines a large cavity size, a high degree of enclosure and framework flexibility in a way that we predicted to enable the encapsulation of larger and more structurally complex guests than is currently possible in discrete abiological systems^22^,^23^.

Cage **1** was stable in acetonitrile, with no degradation observed after 2 months at micromolar concentration under air at 293 K (Supplementary Figs 21–23 and Supplementary Methods). Furthermore, NMR spectroscopy demonstrated **1** to be soluble and stable in a 1:1 mixture of water:acetonitrile (Supplementary Fig. 24), even in the presence of 100 equivalents of 1-methylimidazole, which is a good ligand for Fe^II^ (Supplementary Fig. 25).

### Host–guest studies with small organic molecules

Given the known high affinity of imidazoles for Zn-porphyrins^24^, our studies of the guest-binding properties of **1** focused upon prospective guests containing imidazole moieties. We used 1-methylimidazole (**G1**) as an initial probe, following its binding by ^1^H NMR spectroscopy. Due to the large cavity, only slight upfield shifts of the signals of the β-pyrrolic protons of **1** were observed following the addition of **G1**. In acetonitrile, saturation was observed with only seven equivalents of **G1** (Supplementary Fig. 26). However, when a 1:1 mixture of water:acetonitrile was used, it was necessary to add 70 equivalents to achieve saturation (Supplementary Fig. 27). We infer the weaker interaction in the presence of water to be due to competing hydrogen bonding between water and imidazole.

To quantify affinity constants, ultraviolet–visible titrations were used for subsequent studies. Dissociation constants for the **G1** adduct of **1** were determined to be 30 μM in acetonitrile and 900 μM in 1:1 acetonitrile:water, using a non-cooperative 1:1 porphyrin–imidazole binding model^25^. These findings agreed with the qualitative results of the NMR experiments. Next, we studied the binding of the molecules **G2** and **G3** (Table 1 and Supplementary Figs 13–16) using the same ultraviolet–visible protocol. In acetonitrile the affinity increased slightly with increasing size of the aromatic moiety linked to the imidazole. The affinity for **G3** was thus six times higher than for **G1**. However, this effect is more pronounced in 1:1 acetonitrile:water, where the affinity for **G3** is more than 100 times higher than for **G1**.

Guests **G4** and **G5** (Supplementary Figs 17–20) were prepared in order to further probe the encapsulation properties of **1**. Each has two imidazole moieties connected by a flexible linker that allows binding to either adjoining or opposite zinc porphyrin faces. **G5** contains a naphthalenediimide (NDI) moiety, which tends to stack due to its large, planar aromatic surface^26^, whereas the kinked central linker in **G4** has less tendency to stack. **G5** thus binds to **1** nearly ten times more strongly than **G4** in acetonitrile (Table 1). Remarkably, the difference in affinities was observed to increase more than 300 times in 1:1 acetonitrile:water. Although small, monotopic guests such as **G1** are likely to bind equally well on the inner and outer faces of **1**, the lengths of the multitopic guests would be insufficient to allow them to stretch around the outside of the cube in order to bind to multiple zinc centres at once. The higher binding constants of many of the multitopic guests thus suggest that they bind inside **1**; by doing so they would also release caged solvent molecules in an entropically favourable process. This hypothesis is also supported by fluorescence experiments with **G5**, in which the NDI excimer band was observed after addition of **1** (Supplementary Fig. 47); multiple NDI groups could not stack if bound outside **1**. Attempts at obtaining meaningful diffusion-ordered spectroscopy spectra of the host–guest complexes were unsuccessful due to line broadening.

### Host–guest studies with biomolecules

Following these initial investigations into the potential of **1** for the encapsulation of molecules bearing coordinating groups, we focused our attention on binding biologically relevant guests containing such groups in 1:1 acetonitrile:water. Ritonavir (**G6**) is an antiretroviral medication used to treat HIV/AIDS^27^ that contains two thiazole moieties. The addition of **G6** to a solution of **1** gave changes in the ultraviolet–visible spectra similar to the ones observed for the previous guests. Inferring 3:1 binding within **1**, as with **G5**, a dissociation constant of 104 μM was obtained for **G6**.

Further binding studies were carried out for histidine-containing peptides in 1:1 acetonitrile:water. Histidine, the amino acid that contains an imidazole group in its side chain, is incorporated into a wide variety of pharmacologically relevant peptides. Clavanin A (**G7**) is one such peptide, an antimicrobial agent that is expressed in the hemocytes of the marine organism *Styela Clava*^28^. This drug is used widely as a peptide antibiotic. **G7** consists of 23 amino acids, four of which are histidines, which encouraged us to study its interaction with cage **1**. Remarkably, a dissociation constant of 80 nM was found using a 2:1 binding model, which suggests that each peptide is bound through three histidines to **1**.

Analogous abiological peptide **G8** was also studied, to probe the sequence dependence of the host–guest interaction. **G8** has three histidine residues, one less than **G7**, which are separated by the same number of amino acids as in **G7**. Furthermore, **G8** is also more hydrophilic, containing only five hydrophobic residues, in contrast with the 12 of **G7**. **G8** was observed to bind to **1** with 100 times lower affinity than **G7**, illustrating the importance of secondary interactions. In contrast, peptides without histidines, for example, **G9** and **G10**, were not observed to interact with **1**. These peptides were chosen for their intermediate length between binding peptides **G6** and **G7**/**G8**, to which they are otherwise physicochemically similar.

### Peptide hydrolysis by trypsin in the presence of 1

**Figure 2:**
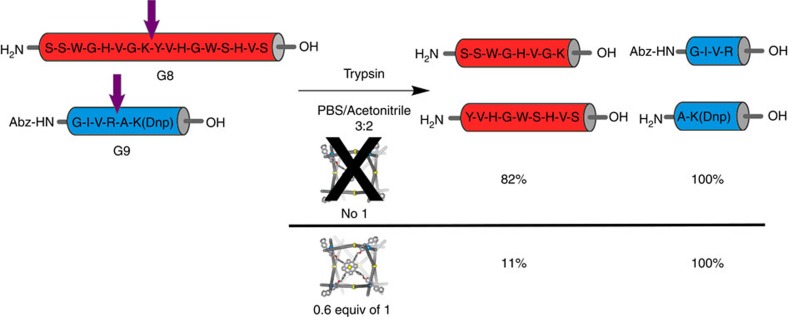
Trypsin treatment of peptides G8 and G9 in the presence and absence of 1. The yield of cleavage for each peptide is shown below the fragments. Arrows indicate the position where peptides are cleaved. Abz, 2-aminobenzoic acid; Dnp, 2,4-dinitrophenyl.

Finally, we envisaged that the closed cubic architecture of **1** could protect its histidine-containing peptide guests from enzymatic degradation. For these experiments we used the protease trypsin, which cleaves exclusively on the C-terminal side of lysine and arginine residues^29^. We initially examined **G7** as a substrate. However, due to its low solubility at neutral pH and its lack of strongly absorbing chromophores, we were unable to follow the reaction by high-performance liquid chromatography (HPLC). Hence, **G8**, which contains two tryptophan chromophores, was used in place of **G7**. The incubation of a 20 μM solution of **G8** with 0.3 equiv. of trypsin in a mixture of acetonitrile and phosphate buffer (pH=7.5) for 30 min led to the cleavage of **G8** in 76% yield (Supplementary Fig. 49). When the same reaction was run in the presence of 0.6 equiv. of **1** (2:1 binding stoichiometry), the extent of cleavage was only 9% (Supplementary Fig. 50). The low degree of enzymatic degradation observed is consistent with the 9 μM dissociation constant of the adduct between **1** and **G8**, which implies the presence of a small amount of free peptide under the reaction conditions. Importantly, when the same experiment was carried out with a non-binding **G9** in place of **G8**, with or without **1**, quantitative **G9** cleavage was observed (Supplementary Figs 52 and 53). In a further test, we treated a mixture of both peptides and **1** with trypsin, and incubated the mixture under identical conditions. In this case almost complete selectivity was observed. Only 11% cleavage occurred for **G8**, whereas **G9** was completely hydrolyzed (Fig. 2, and Supplementary Figs 54 and 55). Importantly, the hydrolysis of **G9** was unaffected by the presence of free **A** or water-soluble Zn-tetrakis(4-sulfonatophenyl)porphyrin (Supplementary Fig. 56), indicating that simple interaction with a zinc porphyrin is insufficient to protect the peptide from the trypsin.

## Discussion

We have thus demonstrated for the first time the utility of a synthetic molecular container for the encapsulation of large and chemically complex biomolecules such as polypeptides and drugs. This became possible as a result of the unique combination of structural features of host **1**, namely: the large-yet-enclosed cavity, the careful balance between rigidity and flexibility of the assembly, and the presence of porphyrinatozinc(II) moieties capable of binding imidazole groups. Moreover, the assembly proved capable of specifically protecting histidine-containing peptides from enzymatic degradation. This work thus represents a crucial step forward in allowing synthetic container molecules to interface with and alter the reactivities of biomolecules. Future work on such molecular systems might enable the development of efficient drug delivery systems or allow for the controlled modulation of biomolecular systems' functioning *in vivo*.

## Methods

### Preparation of **1**

In a J-Young NMR tube porphyrin **A** (4 mg, 3.3 μmol, 6 equiv.; Supplementary Figs 1–4 and Supplementary Methods), iron(II) triflate (1.5 mg, 4.4 μmol, 8 equiv.) and 5,6,7,8-tetrahydronaphthalen-2-amine **B** (1.9 mg, 13 μmol, 24 equiv.) were suspended in [D_7_]DMF (400 μl) and [D_3_]MeCN (100 μl). The solution was deoxygenated by three freeze-pump-thaw cycles, the tube was sealed, and heated in the oil bath for 8 h at 70 °C. After this time, the solution was opened to air and cooled to room temperature. A mixture of diethyl ether (3.5 ml) and chloroform (1.5 ml) was added which resulted in the precipitation of a purple solid. The suspension was centrifuged and the solvent above the solid was removed. The purple solid was washed twice with chloroform (3 ml) and partially dried under nitrogen. The solid was dissolved in acetonitrile (0.5 ml) after drying with nitrogen. **1** (Supplementary Figs 5–12) was not soluble in pure water, but it is soluble in a 1:1 acetonitrile:water mixture (1 mg ml^−1^).

### Ultraviolet–visible absorption spectroscopy

Ultraviolet–visible absorption spectroscopy measurements were performed using a Perkin Elmer Lambda 750 UV-Vis-NIR spectrophotometer fitted with a PTP-1 Peltier temperature controller accessory. Spectra were obtained in double beam mode using only the (front) analyte beam to record spectra, with air in the (rear) reference path. Samples were analysed using quartz cuvettes with optical path lengths of 1 mm. Unless otherwise specified, 417 nm was the wavelength selected to follow the binding of the guest.

### Host–guest chemistry studied by ^1^H NMR

A solution of **1** (0.5 ml) was prepared in a mixture of CD_3_CN:D_2_O (1:1) or in pure CD_3_CN and was transferred to a J-Young NMR tube. Aliquots of the guest were added in the same mixture of solvents. The NMR spectrum was recorded after each addition and the sample was mixed thoroughly before each measurement (Supplementary Figs 26–30).

### Host–guest chemistry studied by ultraviolet–visible spectroscopy

A solution of **1** in CH_3_CN:H_2_O (1:1) (250 μl, 1.5 μM) or pure acetonitrile was transferred to a cuvette. Small aliquots of the guest's solution were titrated into the cuvette. The experiment was performed at room temperature. Each experiment was repeated three times (Supplementary Figs 31–46 and Supplementary Methods). Data obtained from the ultraviolet–visible titration were then fitted using nonlinear analysis with DynaFit programme (Biokin Software)^30^ to the binding equation derived for either 1:1 model (porphyrin:guest with one imidazole group) or 2:1 model (porphyrin:guest with two imidazole groups), except in the case of **G7**. **G7** contains two histidine amino acids in consecutive positions, therefore the distance between the imidazole rings of these two amino acids is too short to allow the interaction with two porphyrins at the same time. In this case, the binding model was 2:1 peptide:cage.

Based on stability experiments, <5% of the cage is degraded during the course of the experiment due to dilution in the mixture of solvents (water/acetonitrile). In these conditions, the right balance is struck between stability of 1 and the accuracy of the affinity constant (Supplementary Tables 1 and 2).

The dilution effect was corrected by multiplying the observe intensity for the dilution factor before the fitting. The error reported for each affinity value is the formal standard error calculated by DynaFit^30^ as the square root of the diagonal elements of the variance–covariance matrix. This s.e. gives an idea of the precision of the fitted value.

### Fluorescence experiments

**G5** fluorescence experiments were carried out with a FS5 Spectrofluorometer, using the following settings: increment: 0.5 nm; integration time: 0.2 s; excitation slit width: 0.5 nm; emission slit width: 0.8 nm at 20 °C. The excitation wavelength was 350 nm and the emission spectra were acquired from 370 to 550 nm. The concentration of **G5** was 1 μM (3 ml) in a mixture of CD_3_CN:D_2_O (1:1). Aliquots of **1** were added in the same mixture of solvents (Supplementary Methods).

### Trypsinization experiments

Trypsinization reactions were followed by analytical RP-HPLC with an Agilent 1,100 series LC using an Eclipse XDB-C18 analytical column (3 × 150 mm, 3.5 μm). Standard conditions for analytical RP-HPLC consisted on a linear gradient from 0 to 10% of solvent B in 5 min, it then increased from 10 to 70% in 30 min at a flow rate of 1.0 ml min^−1^ (A: water with 0.1% trifluoroacetic acid (TFA), B: acetonitrile with 0.1% TFA); 55 μl of solution was injected to the column each time. Compounds were detected by ultraviolet absorption and subsequently characterized by electrospray ionization (ESI) (Supplementary Figs 48–56).

The samples were prepared in 200 μl solution (40% CH_3_CN, 50% phosphate buffer and 10% H_2_O; phosphate buffer: 0.1 M, pH 7.5, 0.2 M NaCl). The concentration of the solution components were: peptides (**G8** and **G9**) 20 μM, trypsin 6 μM and **1** 12 μM. However, not all components were present in each sample, as noted in the main text. The reaction mixtures were stirred at room temperature for 30 min. It was observed that longer reaction times cause degradation of **1** due to the high concentration of NaCl in the phosphate buffer. After this time, H_2_O (200 μl) and acetic acid (8 μl) were added to quench the reaction. In the case of the control experiments with free Zn porphyrins, the concentration of the porphyrin was 72 μM.

### Data availability

Data supporting the findings of this study are available within the article and its Supplementary Information files and from the corresponding author upon reasonable request.

## Additional information

**How to cite this article:** Mosquera, J. *et al*. Sequence-selective encapsulation and protection of long peptides by a self-assembled Fe^II^_8_L_6_ cubic cage. *Nat. Commun.* **8,** 14882 doi: 10.1038/ncomms14882 (2017).

**Publisher's note**: Springer Nature remains neutral with regard to jurisdictional claims in published maps and institutional affiliations.

## Supplementary Material

Supplementary Information Supplementary Figures, Supplementary Tables, Supplementary Methods and Supplementary References

## Figures and Tables

**Table 1:**
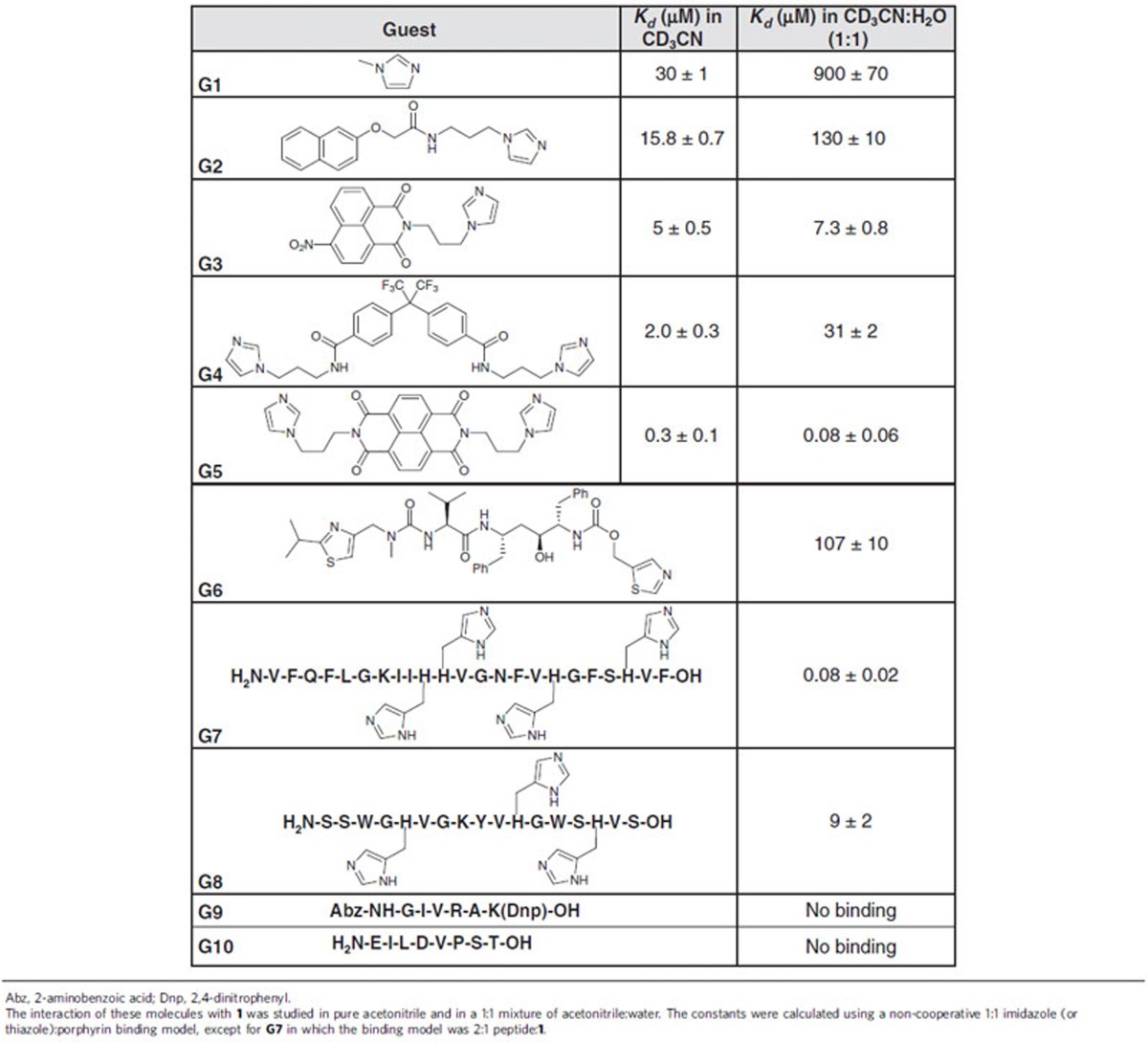
Dissociation constants determined by means of ultraviolet–visible spectroscopy.
