# Supplementary material for: Sequence-selective encapsulation and protection of long peptides by a self-assembled FeII8L6 cubic cage
Source: Nat Commun. 2017 Mar 30;8:14882. doi: 10.1038/ncomms14882 (PMC5379102; doi:10.1038/ncomms14882)
Supplement: Supplementary Information — Supplementary Figures, Supplementary Tables, Supplementary Methods and Supplementary References [file ncomms14882-s1.pdf]

Supplementary Methods

General details:

Chloroform, methanol, *n*-hexane were used without purification. Dichloromethane and pyrrole were distilled over calcium hydride. Other commercial solvents and reagents that were not listed here were acquired from commercial sources and used without further purification. Peptides **G7** and **G8** were purchased from *Genscript* as phosphate salts. **G9** and **G10** were purchased from *Bachem* as trifluoroacetate salts. The acidic protons of the peptides were neutralized through the addition of NaOH before NMR and UV-Vis titrations. 5-Hydroxypicolinaldehyde was purchased from *Fluorochem*. The rest of the reagents were acquired from Sigma-Aldrich.

All reactions were carried out in dry glassware with a nitrogen overpressure. NMR spectra were recorded on a Bruker Advance DPX 400 or Bruker Advance Cryo 500 spectrometer. Chemical shifts for ^{1}H and ^{13}C are reported in ppm on the δ scale; ^{1}H and ^{13}C were referenced to the residual solvent peak. All coupling constants are reported in Hz. Flash column chromatography was performed using Silica Gel high purity grade (pore size 60 Å, 230-400 mesh particle size, Sigma-Aldrich). TLC analyses were performed on Merck TLC Silica Gel 60 F254 Glass plates. Product spots were visualized under UV light (λ_{max} = 254 nm and λ_{max} = 365 nm). All reactions requiring anhydrous conditions were carried out in an oven-dried glassware and under an inert atmosphere of nitrogen. Reaction mixtures were stirred with magnetic stir bars. Centrifugation of samples was carried out using a Grant-Bio LMC-3000 benchtop centrifuge.

Low-resolution electrospray ionisation (ESI) mass spectra were obtained on a Micromass Quattro LC mass spectrometer (cone voltage 15 eV; desolvation temp. 313 K; ionisation temp. 313 K) infused from a Harvard syringe pump at a rate of 10 µL min−1. High-resolution mass spectra were collected at the Department of Chemistry, University of Cambridge, using a ThermoFinnigan Orbitrap Classic mass spectrometer (capillary temp. 30 °C; tube lens 40 V).

DOSY experiments were performed on a Bruker Avance 500 BB ATM spectrometer. Maximum gradient strength was 6.57 G/cm A. The standard Bruker pulse program, ledbpgp2s, employing a stimulated echo and longitudinal eddy-current delay (LED) using bipolar gradient pulses for diffusion using 2 spoil gradients was utilized. Rectangular gradients were used with a total duration of 1.5 ms. Gradient recovery delays were 875-1150 µs. Diffusion times were 100 ms. Individual rows of the S4 quasi-2D diffusion databases were phased and baseline corrected.

Synthesis of ligands:

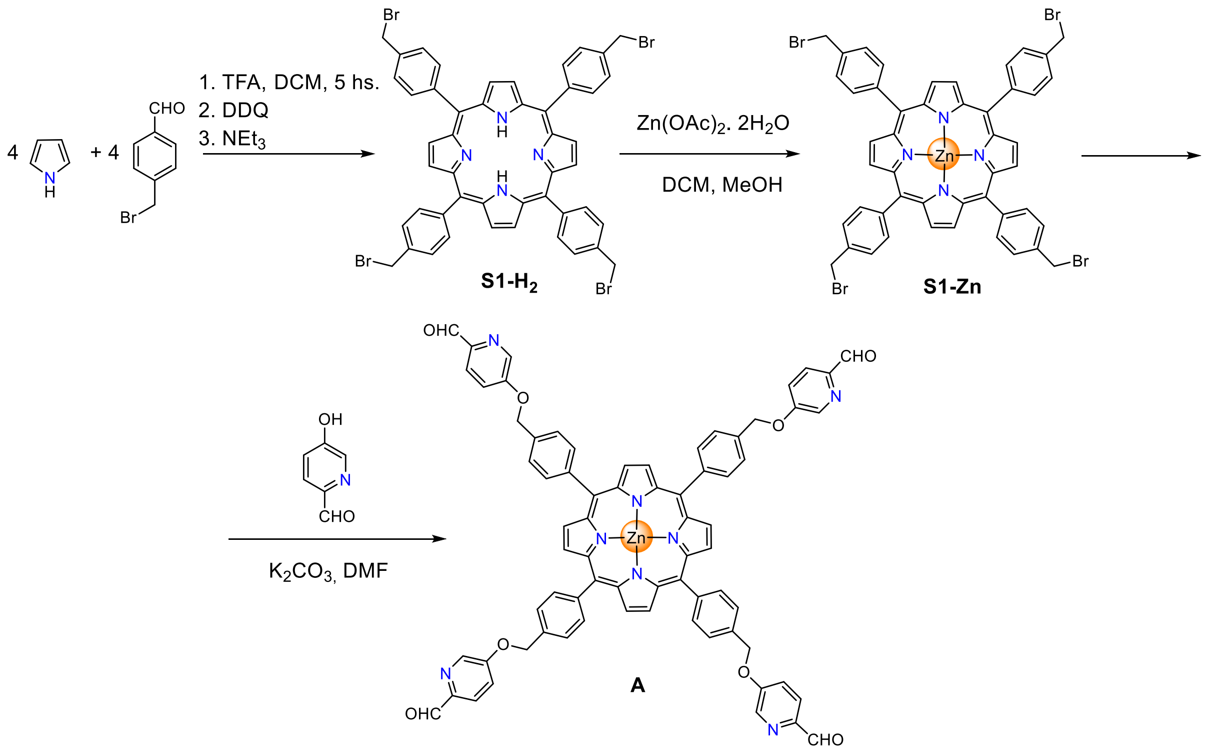

*Tetrakis(4-bromomethylphenyl)porphyrin **S1-H₂***

The compound was obtained following the literature procedure. [1]

4-(Bromomethyl)benzaldehyde (700 mg, 3.5 mmol) and pyrrole (233 μL, 3.5 mmol, 1 equiv.) were dissolved in CH_2Cl_2 (250 mL) and deoxygenated under a stream of N_2 for 15 minutes. Trifluoroacetic acid (210 μL) was added, and the colour of the solution immediately turned golden yellow. The reaction mixture was protected from light and stirred for 5 hours, then DDQ (570 mg, 2.5 mmol) was added and stirring was continued for a further 30 minutes. The reaction was quenched with triethylamine (370 μL) and the crude mixture was passed through a short silica column, flushing with CH_2Cl_2 to remove the purple-red fraction completely. The solvents were removed from the eluate on a rotary evaporator to yield the product as a purple-black microcrystalline solid. Yield: 310 mg (36%).

The analytical data are in agreement with those previously published. [1]

*Synthesis of zinc(II) tetrakis(4-bromomethylphenyl)porphyrin **S1-Zn**.*

Tetrakis(4-bromomethylphenyl)porphyrin **S1-H₂** (187 mg, 0.2 mmol) and zinc(II) acetate dihydrate (500 mg, 2.3 mmol, 14 equiv.) were dissolved in a mixture of dichloromethane (30 mL) and methanol (10 mL). The solution was stirred for 4 hours. The solution was transferred to a separating funnel and washed three times with distilled water (3x200 mL) and once with brine (100 mL). Organic phase was collected, dried over anhydrous sodium sulphate and filtrated. Evaporation of the solvent yielded pure product as a red-purple solid. Yield: 190 mg (95%).

^{1}**H NMR** (400 MHz, $CDCl_3$, 298 K): δ (ppm) 8.94 (s, 8H), 8.19 (d, $^{3}J_{HH}$ = 8.0 Hz, 8H), 7.79 (d, $^{3}J_{HH}$ = 8.0 Hz, 8H), 4.87 (s, 8H).

^{13}**C NMR** (100 MHz, $CDCl_3$, 298 K): *δ* (ppm) 150.2(C), 143.0(C), 137.3(C), 134.9(CH), 132.2(CH), 127.5(CH), 120.7(C), 33.8(CH_2).

HRMS (ESI+, TOF) *m/z* $[M+H]^+$ 1044.8683, calcd: $[C_{48}H_{33}N_4{}^{79}Br_4Zn_1]^+$ 1044.8725.

See Supplementary Figs. 1 and 2 for experimental data.

Synthesis of zinc(II) tetrakis(4-(((6-formylpyridin-3-yl)oxy)methyl)-phenyl)porphyrin ***A***

In a 2 mL round-bottomed flask 5-hydroxypicolinaldehyde (18 mg, 0.15 mmol) and potassium carbonate (25 mg) were placed. The flask was evacuated and refilled with nitrogen three times. Dry DMF (0.5 mL) was added under nitrogen. The mixture was heated to 70℃ and zinc(II) tetrakis(4-bromomethylphenyl)porphyrin **1-Zn** (30 mg, 0.03 mmol) was added. The reaction mixture was heated at 70℃ for 12 hours. After this time the mixture was cooled down and diluted with water (5 mL) which was accompanied by precipitation of the crude product. The suspension was centrifuged, solution above the solid was removed and this procedure was repeated three times (3 x 3 mL of water) in order to remove residual DMF and potassium carbonate. The product was dried under vacuum. Yield: 28 mg (80%).

^{1}**H NMR** (400 MHz, $[D_7]$DMF, 298 K): δ (ppm) 10.02 (s, 4H), 8.95 – 8.88 (s, 8H), 8.82 (d, $^{4}J_{HH}$ = 2.7 Hz, 4H), 8.36 (d, $^{3}J_{HH}$ = 7.9 Hz, 8H), 8.12 (d, $^{3}J_{HH}$ = 8.6 Hz, 4H), 8.04 – 8.00 (d overlapped with solvent signal, 8H), 7.97 (dd, $^{3}J_{HH}$ = 8.6 Hz, $^{4}J_{HH}$ = 2.7 Hz, 4H), 5.78 (s, 8H).

^{13}**C NMR** (100 MHz, $[D_7]$DMF, 298 K): *δ* (ppm) 193.1(CH), 159.8(C), 151.0(C), 147.7(C), 144.3(C), 140.4(CH), 136.6(C), 135.9(CH), 132.7(CH), 127.5(CH), 124.4(CH), 122.8(CH), 121.4(C), 71.7(CH_2).

HRMS (ESI+, TOF) *m/z* $[M+Na]^+$ 1239.2758, calcd: $[C_{72}H_{48}N_8NaO_8Zn]^+$ 1239.2784.

See Supplementary Figs. 3 and 4 for experimental data.

Preparation of **1**:

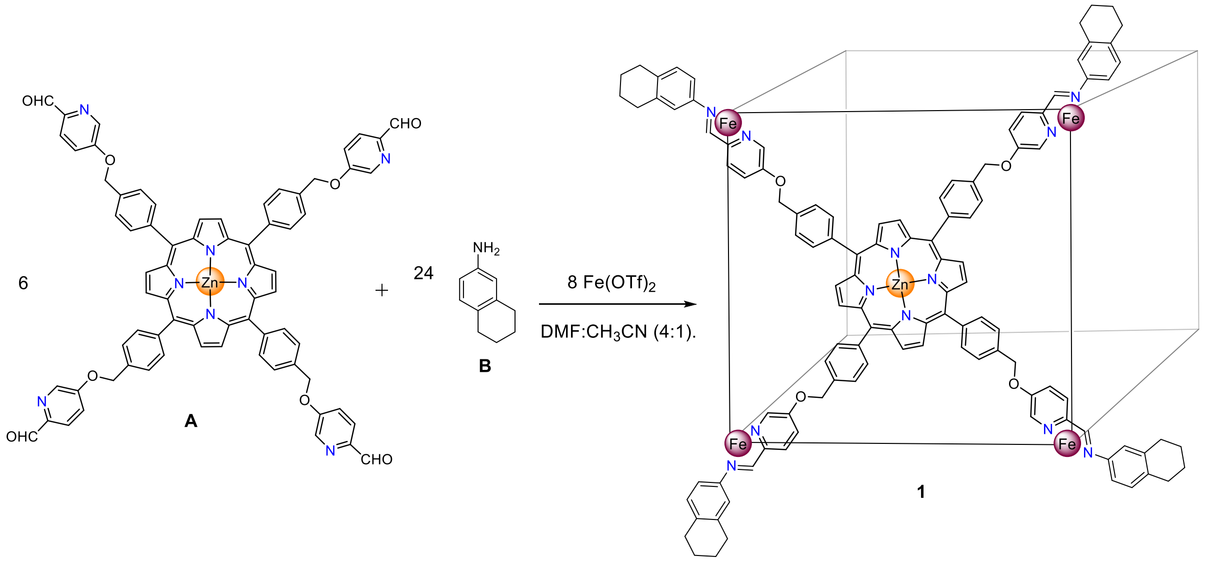

Synthesis in methods section of main text.

The assembly should not be dried completely, otherwise it is not possible to re-dissolve it. The reason could be that filling the cavity with solvent is a kinetically slow process.

^{1}H NMR (400 MHz, CD_3CN, 298 K) δ 8.74 (s, 48H), 8.71 (s, 24H), 8.61 (d, $^3J_{HH}$ = 8.7 Hz, 24H), 8.32 (d, $^3J_{HH}$ = 8.7 Hz, 24H), 8.27 (d, $^3J_{HH}$ = 7.6 Hz, 24H), 8.02 (d, $^3J_{HH}$ = 7.6 Hz, 24H), 7.90 (d, $^3J_{HH}$ = 7.6 Hz, 24H), 7.84 (d, $^3J_{HH}$ = 7.6 Hz, 24H), 7.29 (s, 24H), 6.99 (d, $^3J_{HH}$ = 8.2 Hz, 24H), 5.72 (d, $^2J_{HH}$ = 8.9 Hz, 24H), 5.54 (d, $^2J_{HH}$ = 8.9 Hz, 24H), 5.36 (d, $^3J_{HH}$ = 8.2 Hz, 24H), 4.97 (s, 24H), 2.80 (broad peak, 48 H), 2.56 (broad peak, 48H), 1.83 (broad peak, 96H).

^{13}C NMR (100 MHz, CD_3CN, 298 K) *δ* (ppm) 173.1(CH), 160.0(C), 152.2(C), 150.7(C), 150.5(C), 148.9(C), 148.3(CH), 145.3(C), 139.4(C), 138.5(C), 135.4(CH), 135.3(CH), 134.8(C), 132.7(CH), 132.6(CH), 132.5(CH), 130.6(CH), 129.5(CH), 122.8(CH), 122.0(CH), 119.6 (CH), 73.2(CH_2), 30.3(CH_2), 29.7(CH_2), 23.8(CH_2), 23.7(CH_2).

See Supplementary Figs. 5 and 12 for experimental data.

Synthesis of guests:

Synthesis of ***G2***

2-Naphthoxyacetic acid (150 mg, 0.7 mmol) reacted with HATU (1-[bis(dimethylamino)methylene]-1H-1,2,3-triazolo[4,5-b] pyridinium 3-oxide hexafluorophosphate, 258 mg, 0.7 mmol) in the presence of N,N-diisopropylethylamine (260 µL) and DMF (3 mL). The mixture was stirred for 5 minutes at room temperature. 1-(3-Aminopropyl)imidazole (734 µL, 6.2 mmol, 8.8 equiv.) was added to the reaction mixture and was stirred overnight at room temperature. The solution was concentrated under reduced pressure and purified by column chromatography in MeOH/DCM (0-15%). Yield: 158 mg (69%).

^{1}**H NMR** (400 MHz, $CDCl_3$, 298 K) δ 7.79 (d, $^{3}J_{HH}$ = 8.0, 1H), 7.78 (d, $^{3}J_{HH}$ = 6.4, 1H), 7.74 (d, $^{3}J_{HH}$ = 8.1, 1H), 7.59 (s, 1H), 7.47 (dd, $^{3}J_{HH}$ = 8.1, 6.9 Hz, 1H), 7.38 (dd, $^{3}J_{HH}$ = 6.9, 6.4 Hz, 1H), 7.21 (dd, $^{3}J_{HH}$ = 8.0 Hz, $^{4}J_{HH}$ = 2.5 Hz, 1H), 7.14 (d, $^{4}J_{HH}$ = 2.5 Hz, 1H), 7.06 (s, 1H), 6.92 (s, 1H), 6.74 (broad peak, 1H), 4.62 (s, 2H), 3.98 (t, $^{3}J_{HH}$ = 6.9 Hz, 2H), 3.39 (t, $^{3}J_{HH}$ = 6.6 Hz, 2H), 2.07 (tt, $^{3}J_{HH}$ = 6.9, 6.6 Hz, 2H).

^{13}**C NMR** (100 MHz, $CDCl_3$, 298 K) *δ* (ppm) 168.8(C), 155.0(C), 137.1(CH), 134.4(C), 130.2(CH), 129.6(C), 128.6(CH), 127.9(CH), 127.1(CH), 127.0(CH), 124.6(CH), 119.2(CH), 118.2(CH), 107.6(CH), 67.4(CH_2), 44.9(CH_2), 36.4(CH_2), 31.1(CH_2).

HRMS (ESI+, TOF) *m/z* $[M+H]^+$ 310.1540, calcd: $[C_{17}H_{17}N_3O_2]^+$ 310.1550.

See Supplementary Figs. 13 and 14 for experimental data.

Synthesis of ***G3***

4-Nitro-1,8-naphthalic anhydride (181 mg, 0.7 mmol) and 1-(3-aminopropyl)imidazole (98 µL, 0.8 mmol, 1.1 equiv.) were dissolved in DMF (2 mL). The reaction was completed under microwave synthesis at 100℃ for 30 minutes. The reaction mixture was extracted with CH_2Cl_2 and washed with water three times. The organic phase was collected, dried over anhydrous magnesium sulphate and filtrated. Evaporation of the solvent yielded pure product as a white solid. Yield: 184 mg (71%).

^{1}**H NMR** (400 MHz, $CDCl_3$, 298 K) δ 8.86 (d, $^{3}J_{HH}$ = 8.7, 1H), 8.76 (d, $^{3}J_{HH}$ = 8.5, 1H), 8.71 (d, $^{3}J_{HH}$ = 8.0, 1H), 8.41 (d, $^{3}J_{HH}$ = 8.0, 1H), 8.02 (dd, $^{3}J_{HH}$ = 8.5, 8.7, 1H), 7.57 (s, 1H), 7.03 (s, 1H), 7.01 (s, 1H), 4.29 (t, $^{3}J_{HH}$ = 7.4, 2H), 4.10 (t, $^{3}J_{HH}$ = 7.3, 2H), 2.24 (tt, $^{3}J_{HH}$ = 7.4, 7.3 Hz, 2H)

^{13}**C NMR** (100 MHz, $CDCl_3$, 298 K) *δ* (ppm) 163.5(C), 162.7(C), 149.9(C), 137.3(CH), 132.8(CH), 130.2(CH), 130.2(CH), 129.8(CH), 129.7(CH) 129.2(C), 126.7(C), 124.1(C), 123.9(CH), 122.8(C), 118.8(CH), 45.1(CH_2), 38.3(CH_2), 29.5(CH_2).

HRMS (ESI+, TOF) *m/z* $[M+H]^+$ 351.1080, calcd: $[C_{18}H_{14}N_4O_4]^+$ 351.1088.

See Supplementary Figs. 15 and 16 for experimental data.

Synthesis of ***G4***

4,4′-(Hexafluoroisopropylidene)bis(benzoic acid) (100 mg, 0.3 mmol) was reacted with HATU (213 mg, 0.6 mmol) in the presence of N,N-diisopropylethylamine (177 µL) and DMF (2.5 mL). The mixture was stirred for 5 minutes at room temperature. 1-(3-Aminopropyl)imidazole (82 µL, 0.7 mmol, 2.7 equiv.) was added to the reaction mixture and was stirred overnight at room temperature. Water (20 mL) was added, and the resulting crude reaction mixture was diluted with AcOEt (5 mL) and transferred to a separation funnel. The organic layer was separated and dried with $MgSO_4$, filtered and the solvent removed under reduced pressure. The solution was concentrated under reduced pressure and purified by column chromatography in MeOH/DCM (0-20%). Yield: 77 mg (50%).

^{1}**H NMR** (400 MHz, MeOD, 298 K) δ 7.91 (d, $^{3}J_{HH}$ = 8.3 Hz, 4H), 7.88 (s, 2H), 7.73 (s, 2H), 7.50 (d, $^{3}J_{HH}$ = 8.3 Hz, 4H), 7.20 (s, 2H), 6.99 (s, 2H), 4.14 (t, $^{3}J_{HH}$ = 6.6 Hz, 4H), 3.43 (t, $^{3}J_{HH}$ = 6.8 Hz, 4H), 2.14 (tt, $^{3}J_{HH}$ = 6.6 Hz, 6.8 Hz, 4H).

^{13}**C NMR** (100 MHz, MeOD, 298 K) *δ* (ppm) 169.2(C), 138.5(CH), 137.3(C), 136.7(C), 131.4(CH), 129.1(CH), 128.5(CH), 120.7 (CH), 79.5 (C), 65.9 (C), 45.7(CH_2), 38.3(CH_2) 31.9(CH_2).

HRMS (ESI+, TOF) *m/z* $[M+H]^+$ 607.2233, calcd: $[C_{29}H_{28}N_6O_2F_6]^+$ 607.2251.

See Supplementary Figs. 17 and 18 for experimental data.

Synthesis of ***G5***

1,4,5,8-Naphthalenetetracarboxylic dianhydride (100 mg, 0.4 mmol) and 1-(3-aminopropyl)imidazole (89 µL, 0.7 mmol, 2 equiv.) were dissolved in DMF (2 mL). The reaction was completed under microwave synthesis at 150℃ for 1 hour. The reaction mixture was extracted with CH_2Cl_2 and washed with water three times. The organic phase was collected, dried over anhydrous magnesium sulphate and filtrated. Evaporation of the solvent yielded pure product as a golden solid. Yield: 154 mg (86%).

^{1}**H NMR** (400 MHz, $CDCl_3$, 298 K) δ 8.76 (s, 4H), 7.53 (s, 2H), 6.99 (s, 2H), 6.98 (s, 2H) 4.27 (t, $^{3}J_{HH}$ = 7.0 Hz, 4H), 4.12 (t, $^{3}J_{HH}$ = 7.2 Hz, 4H), 2.30 (tt, $^{3}J_{HH}$ = 7.2, 7.0 Hz, 4H).

^{13}**C NMR** (100 MHz, $CDCl_3$, 298 K) *δ* (ppm) 163.0(C), 137.3(CH), 131.3(CH), 129.9(CH), 126.8(C), 126.6(C), 118.7(CH), 45.1(CH_2), 38.5(CH_2), 29.3(CH_2).

HRMS (ESI+, TOF) *m/z* $[M+H]^+$ 483.1763, calcd: $[C_{26}H_{22}N_6O_4]^+$ 483.1775.

See Supplementary Figs. 19 and 20 for experimental data.

<u>Stability of **1**:</u>

Cage **1** showed very high stability in acetonitrile; no degradation was observed even after several weeks at micromolar concentration (Supplementary Fig. 21). Encouraged by this observation, we hypothesized that **1** might be stable enough to be used in a mixture of acetonitrile and water. Assemblies based on imine-bond formation have been observed to be most stable in water when the subcomponents are also water-soluble. When subcomponents are insoluble, the small equilibrium population of insoluble free subcomponents precipitate, driving the equilibrium towards decomposition of the cage. Remarkably, although subcomponent **A** was not observed to dissolve in either water or acetonitrile, NMR spectroscopy demonstrated **1** to be soluble and stable in a 1:1 mixture of water:acetonitrile. No degradation of a 80 µM solution of **1** in water:acetonitrile was observed after 24 h (Supplementary Fig. 24), even in the presence of 100 equivalents of 1-methylimidazole (Supplementary Fig. 25), which is a good ligand for Fe^{II}. However, a small degree of degradation was observed at lower concentrations (Supplementary Fig. 22). Around 5% of the UV-Vis signal intensity was lost after one hour at 1.5 µM concentration.

Host-guest model:

The experimental data obtained using UV-Vis spectroscopy for each guest was fitted using nonlinear analysis with DynaFit program (Biokin Software)[2] to the binding equation derived for 1:1 model porphyrin:imidazole group (non-cooperative model). This model is ideal for **G1** (1-methylimidazole), which is so small that the binding to one porphyrin does not affect the interaction with the rest of porphyrins present in **1**. Therefore, each porphyrin of **1** must bind to one **G1** molecule in the saturation point, with the six affinity constants having the same value. The Kd value was 30 µM in acetonitrile, consistent with values obtained in previous studies [3].

The decrease in absorption at 417 nm observed in the case of **G1** was also observed in the cases of the other guests. As shown in the Supplementary Tables 1 and 2, the behavior observed was similar for all guests. We thus inferred that in all cases each porphyrin unit of **1** was interacting with one imidazole group in the saturation point of the titration. In this way, a non-cooperative model was used to obtain the intrinsic dissociation constant for the rest of the guests[4].

Host-guest chemistry studied by fluorescence spectroscopy:

In order to test our hypothesis that guest is interacting with the zinc porphyrins from within the cavity of **1**, we decided to exploit the fluorescence properties of NDI moieties present in **G5**. The NDI exhibits aggregation-induced emission enhancement in water solutions [5]. Therefore, if three NDI units are stacked on top of each other in the cavity of the cage, as we foresaw for G**5**, an increase in the fluorescence signal of the NDI should be observed, accompanied by the appearance of the excimer band, as in the aggregation process. Indeed, when a solution of **G5** was added to **1**, the expected changes in the spectra were observed. The NDI fluorescence increased, and a new excimer band appeared at 470 nm. The solution was saturated with around 0.35 equivalents of the cage, as expected from the Kd of the ligand. The model (Supplementary Fig. 47) showing the interactions between the host and the guest molecules was prepared using the CAChe Workspace [6]. The MM2-optimized structure of the complex anticipates that the NDI moieties of the guests form a stack of three rings through aromatic stacking interactions.

Volume calculations

In order to determine the available void space within the **1**, VOIDOO [7] calculations based on the optimized structures were performed. A virtual probe with the minimum radius such that it would not exit the cavity during the calculation was employed. The following parameters were changed from their default values:

Probe radius	4 Å
Maximum number of volume-refinement cycles:	30
Minimum size of secondary grid:	3

Grid for plot files: 0.2

Primary grid spacing: 0.2

The flexibility of **1** was determined through the expansion and contraction of the structure (See Supplementary Fig. 57 for normal structure). In order to obtain the expanded structure, the positive charge of the zinc atoms present in the porphyrins was increased to +5. In this way, the high electrostatic repulsion between the porphyrins and the irons of the corner leads to the expansion of the cage after an energy minimization with MM2 level of theory in CAChe Workspace. Then, the charge of zinc atoms was returned to the correct value (+2), and the energy minimization was repeated (*5*) (Supplementary Fig. 58).

The contracted conformation was achieved using the following protocol: Firstly, porphyrins in opposite faces were connected through coordination with 4,4'-bipyridine. The structure was energy minimized. 4,4'-bipyridine was removed from the structure. Finally, the minimization step was repeated (Supplementary Fig. 59).

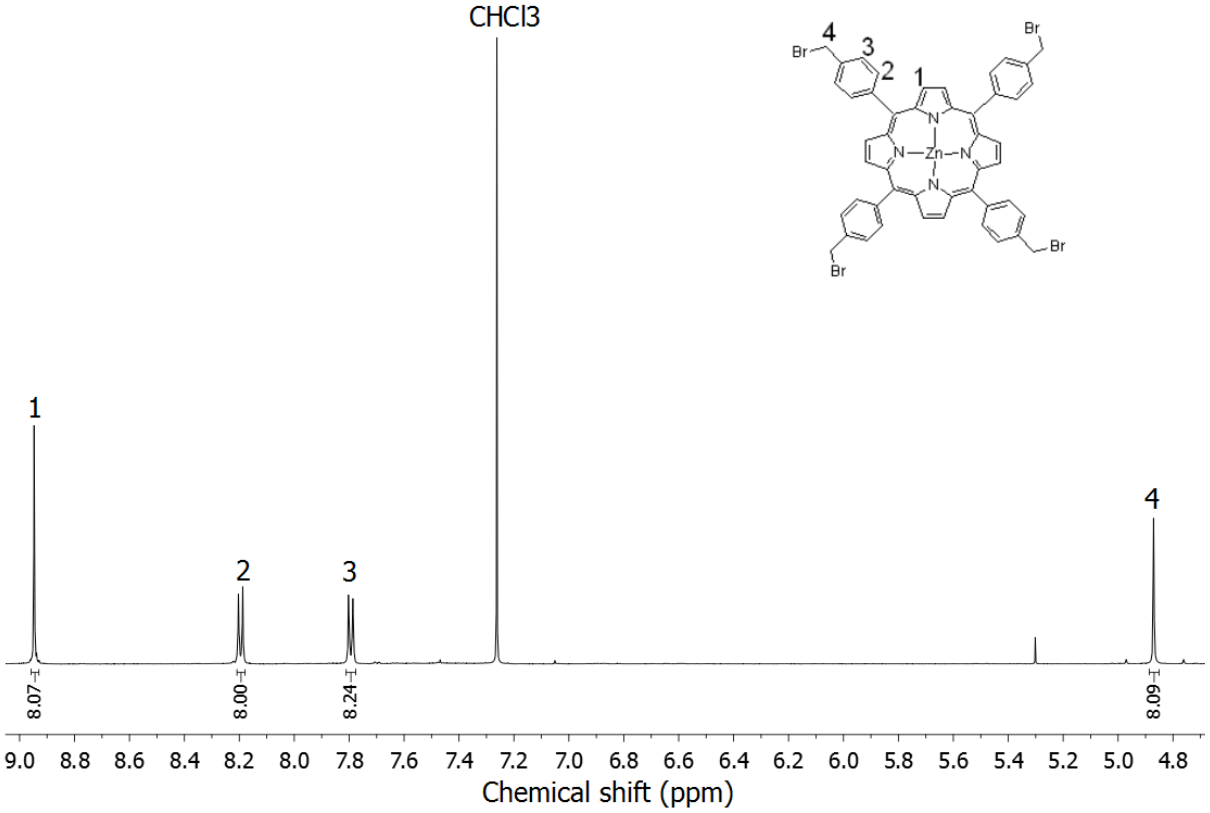

Supplementary Figure 1 | ^{1}H NMR spectrum of S1-Zn (400 MHz, $CDCl_3$, 298 K).

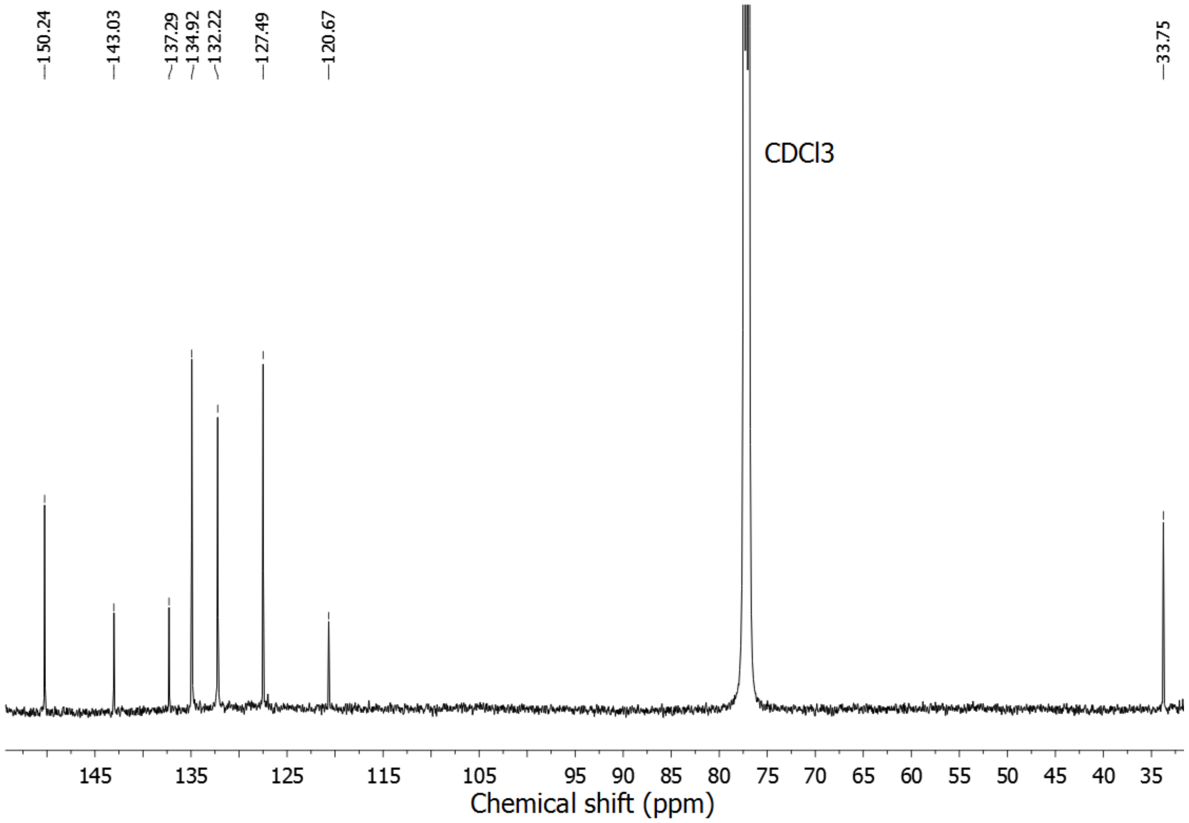

Supplementary Figure 2 | ^{13}C NMR spectrum of S1-Zn (100 MHz, $CDCl_3$, 298 K).

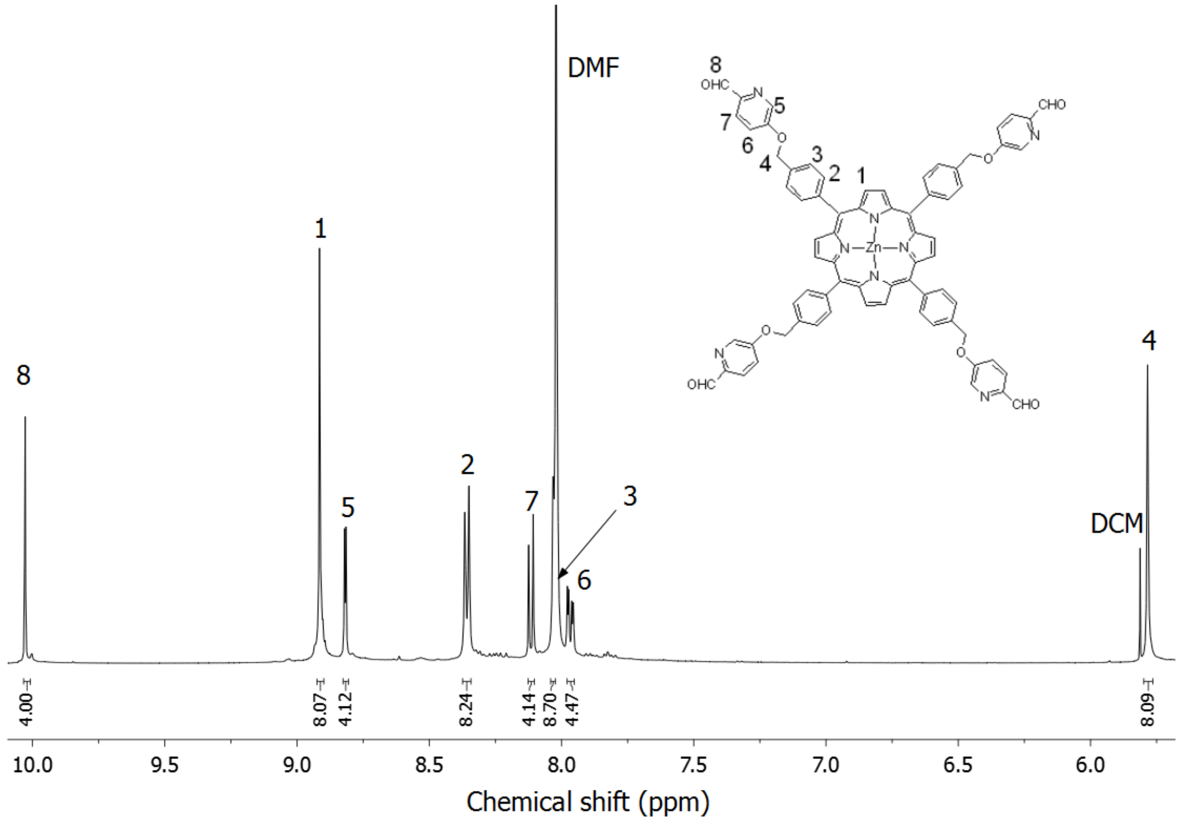

Supplementary Figure 3 | ^{1}H NMR spectrum of A (400 MHz, [D_7]DMF, 298 K).

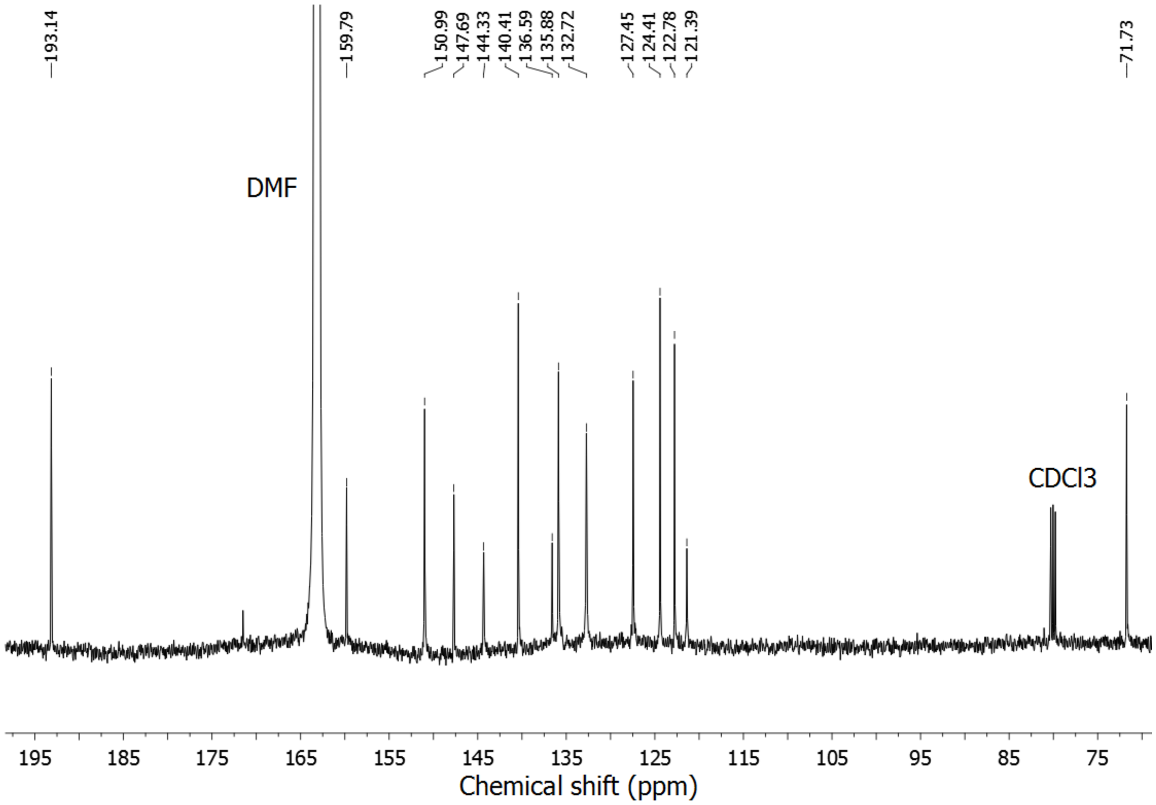

Supplementary Figure 4 | ^{13}C NMR spectrum of A (100 MHz, [D$_7$]DMF, 298 K).

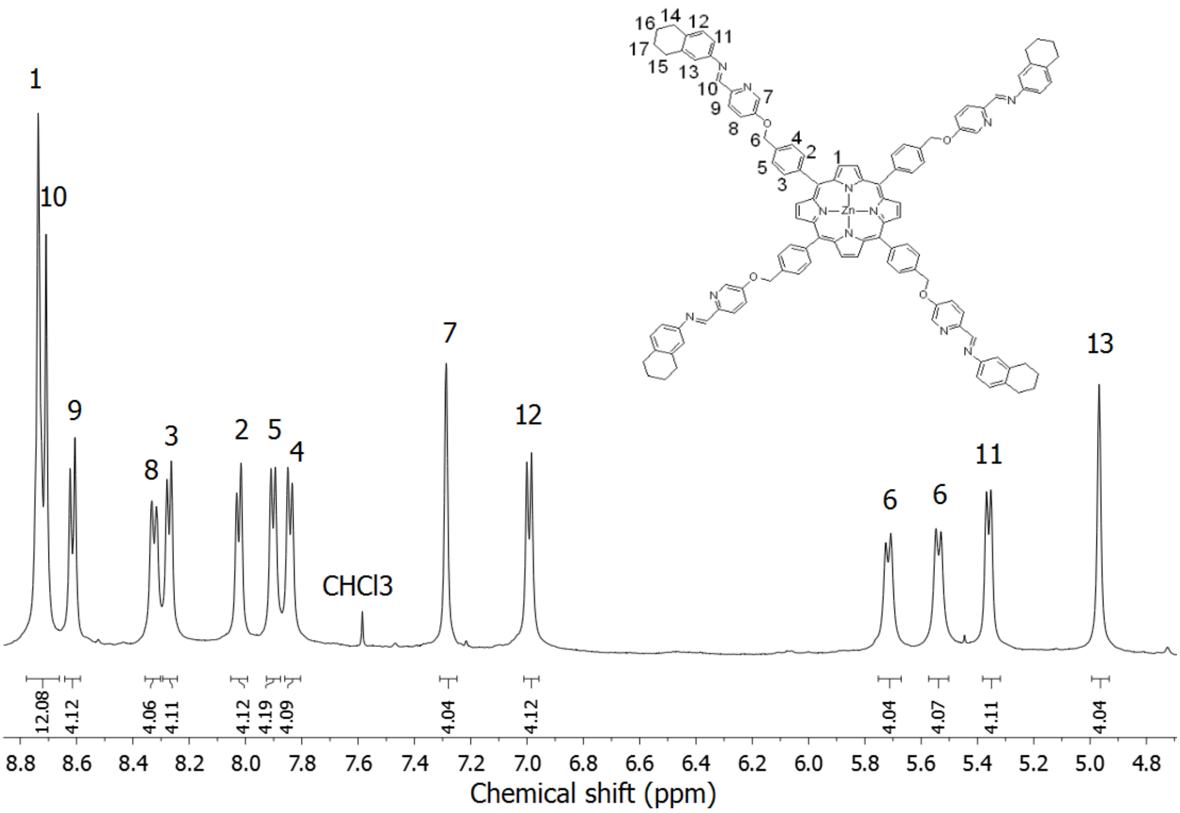

Supplementary Figure 5 | ^{1}H NMR spectrum of 1 (400 MHz, CD_3CN, 298 K). Protons 2 and 4 were pointing towards the cavity of the cage. Protons 3 and 5 were pointing out of the cage.

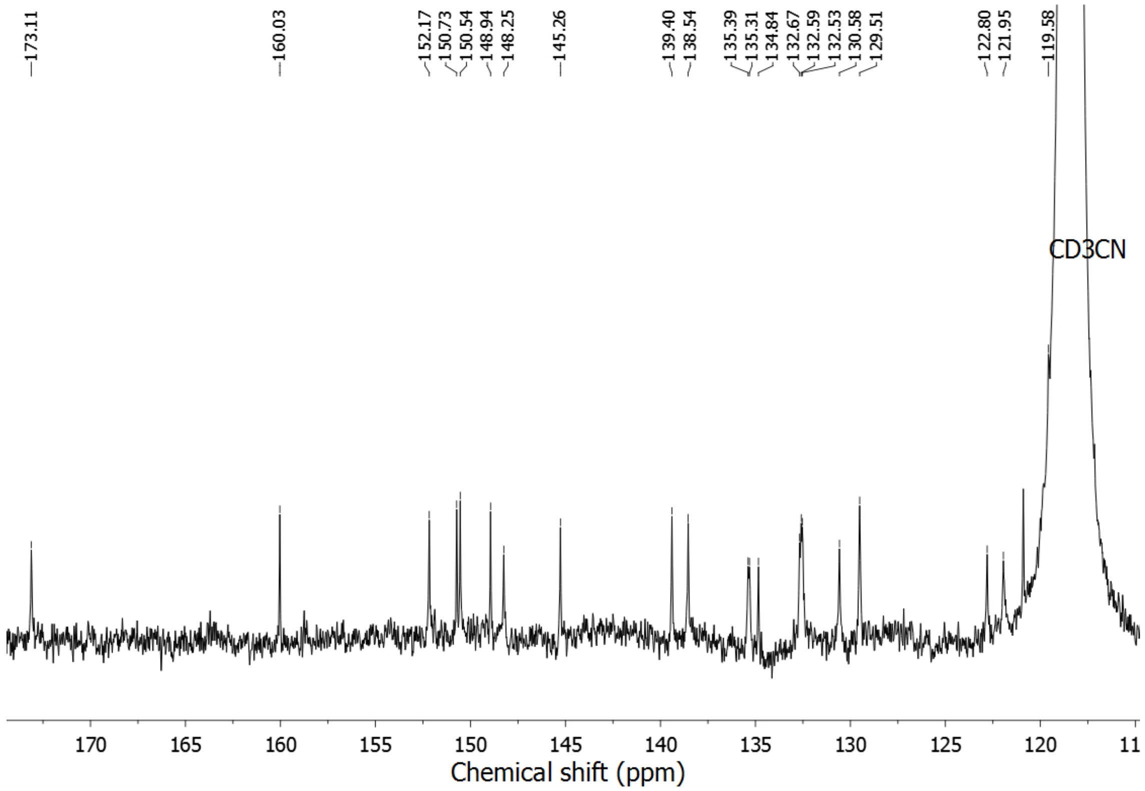

Supplementary Figure 6 | ^{13}C NMR spectrum of 1 (100 MHz, CD_3CN, 298 K).

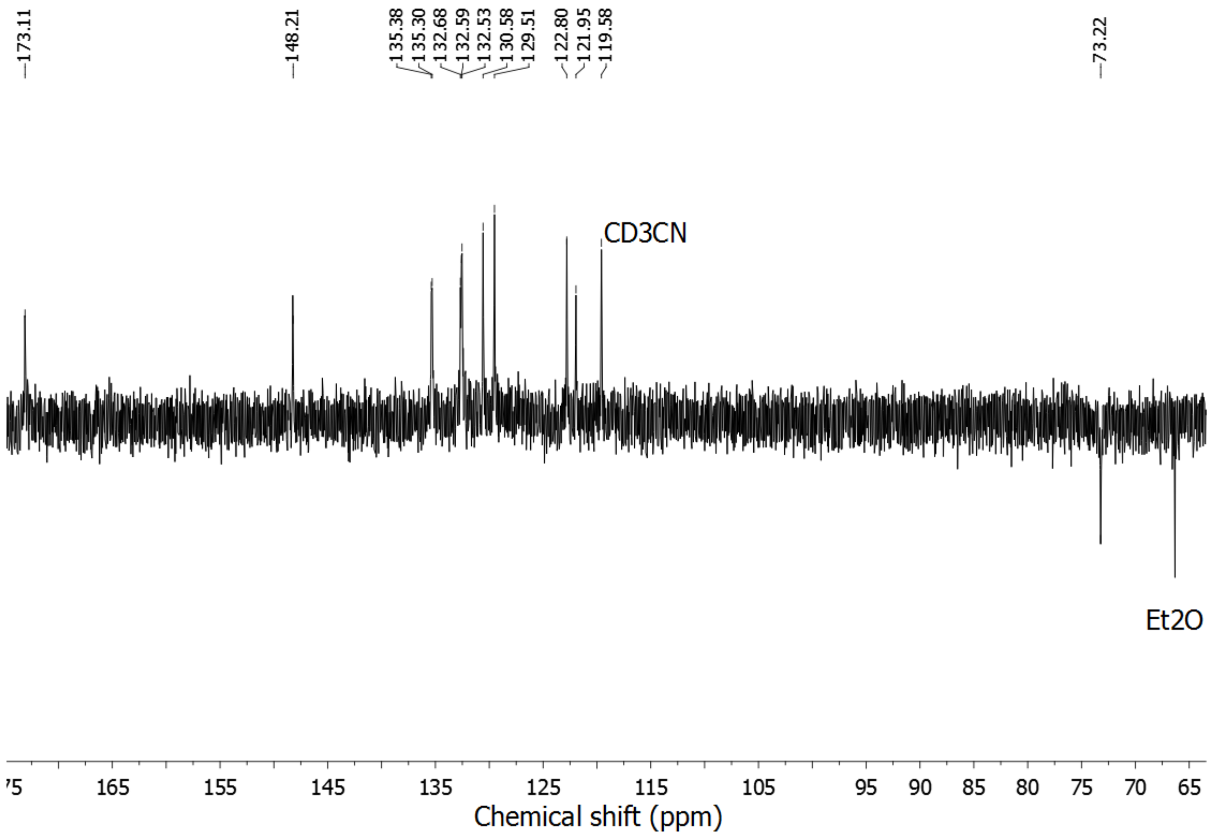

Supplementary Figure 7 | Aromatic region of ^{13}C DEPT spectrum of 1 (100 MHz, CD_3CN, 298 K).

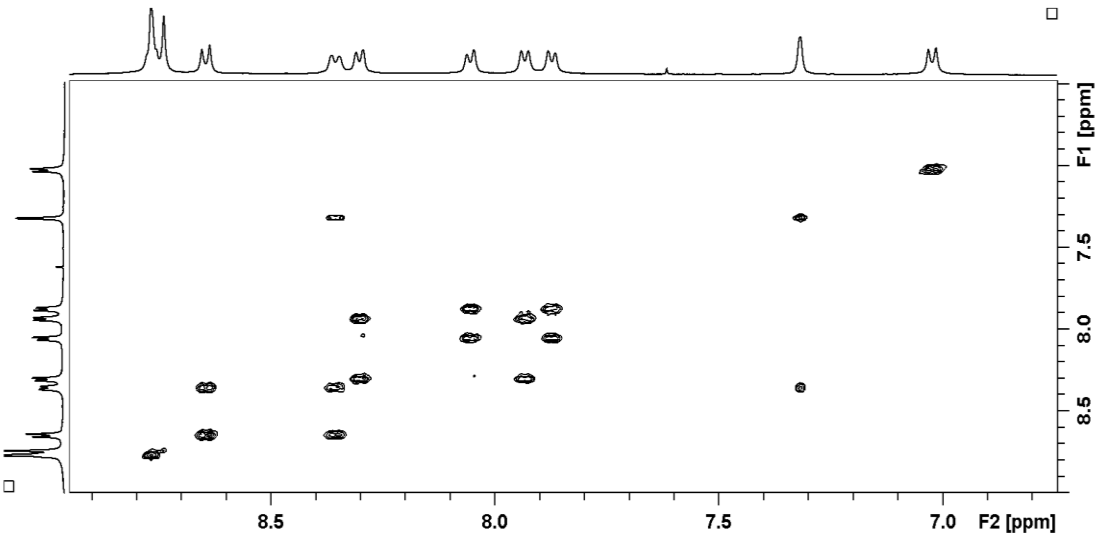

Supplementary Figure 8 | Aromatic region of ^1H-^1H COSY spectrum of 1 (500 MHz, CD_3CN, 298 K).

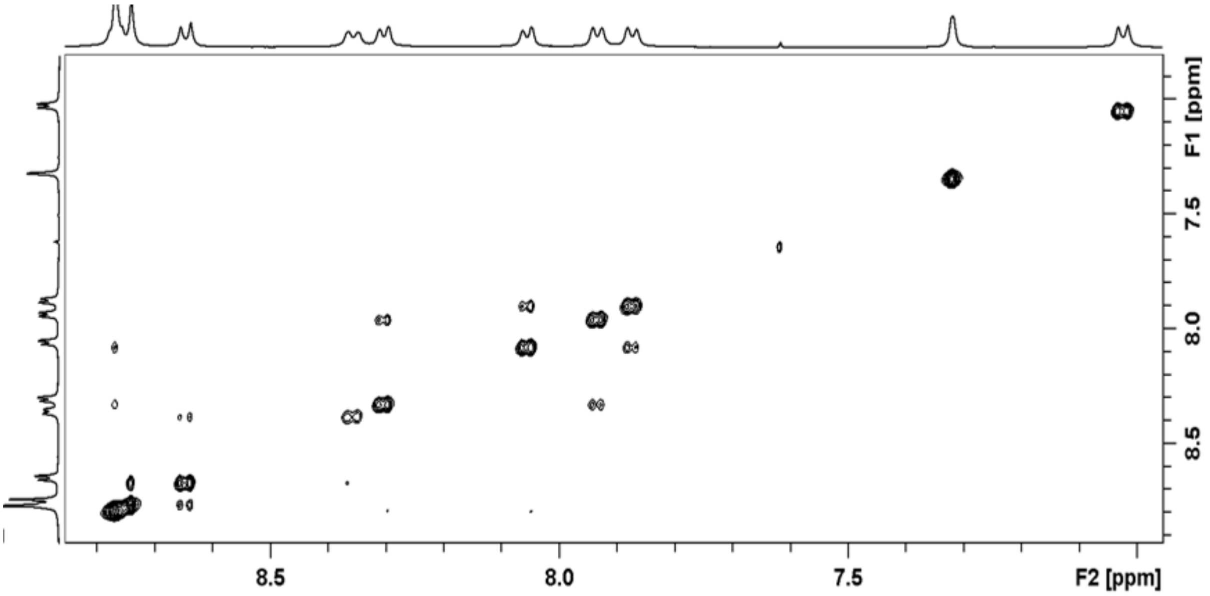

Supplementary Figure 9 | Aromatic region of ^1H-^1H NOESY spectrum of 1 (500 MHz, CD_3CN, 298 K).

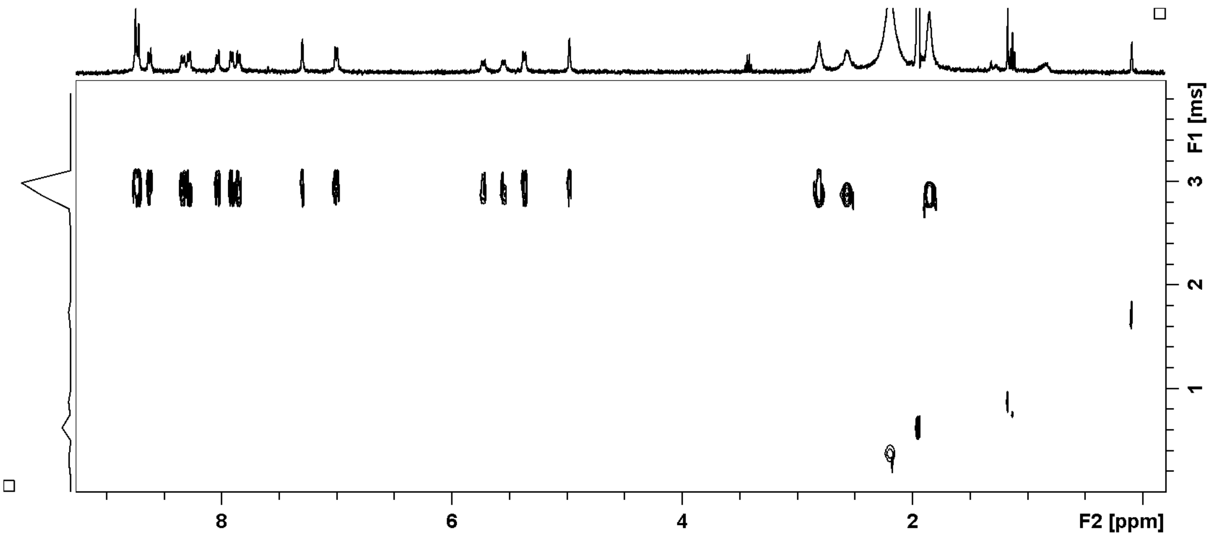

Supplementary Figure 10 | DOSY spectrum of 1 (500 MHz, CD_3CN, 298 K). The diffusion coefficient equals to 2.81×10^{-10} m^2/s. The hydrodynamic radius is of 19.7 Å, which is consistent with the range of 21 – 17 Å derived from the different models of **1**. The theoretical radius was obtained as the mean of two different distances from the MM2 energy-minimized models: the distance between opposite Zn^{II} centers and the distance between the furthest hydrogen atoms of opposite anilines.

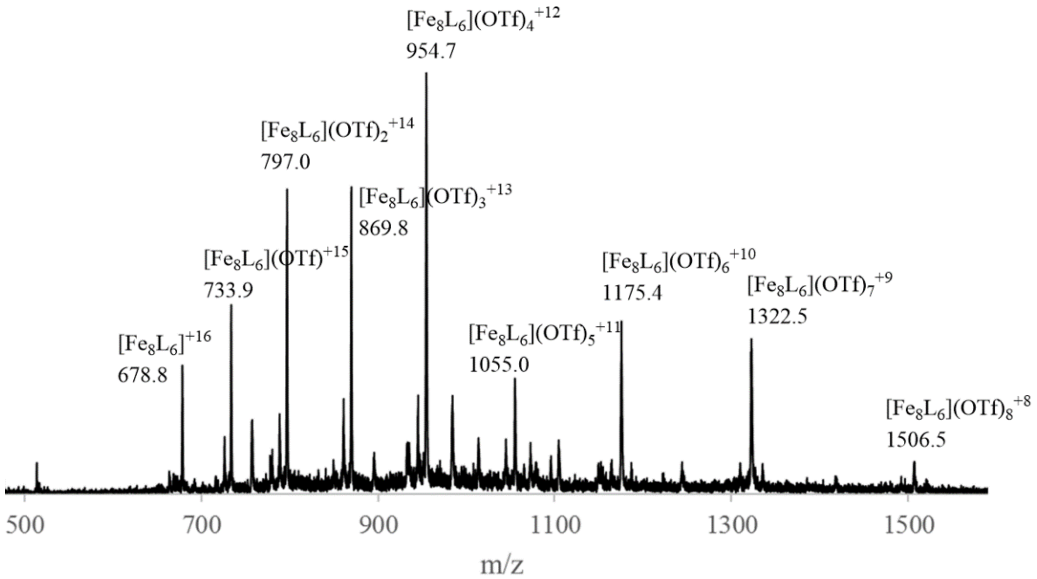

Supplementary Figure 11 | Low-resolution ESI mass spectrum of 1.

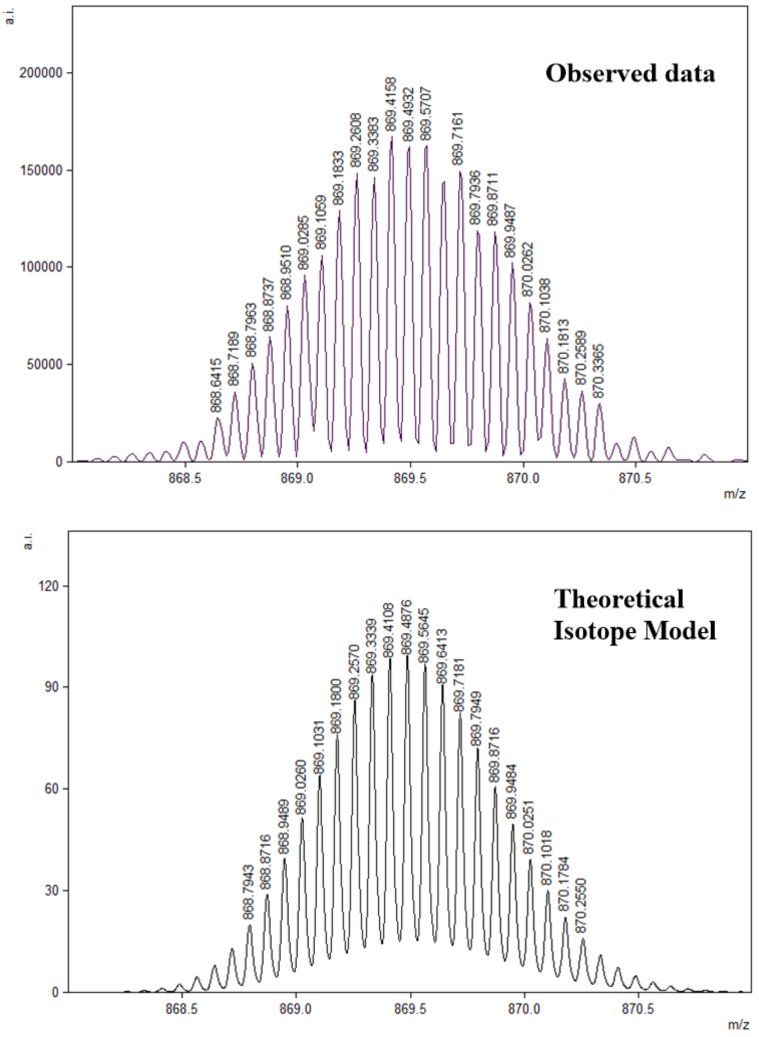

Supplementary Figure 12 | High-resolution ESI-mass spectrum of 1 showing +13 peak.

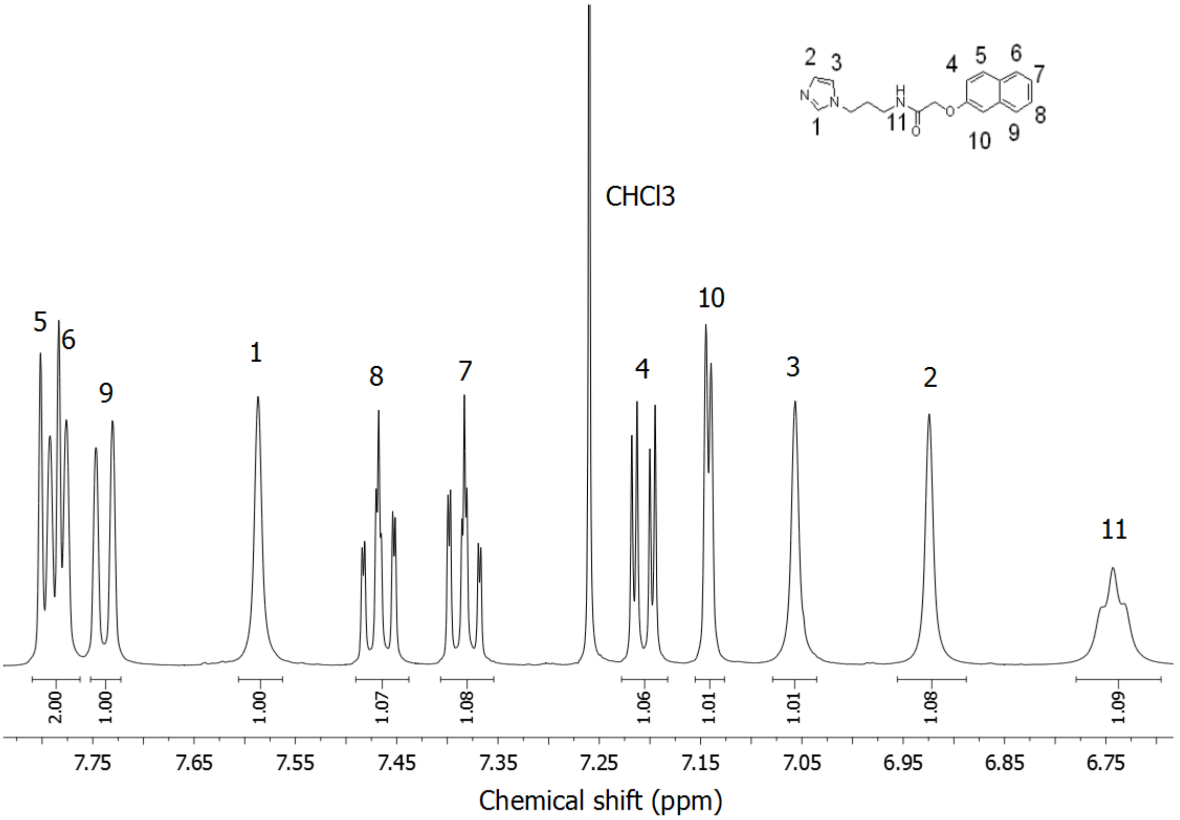

Supplementary Figure 13 | ^{1}H NMR spectrum of G2 (400 MHz, $CDCl_3$, 298 K). The aliphatic region was omitted for clarity.

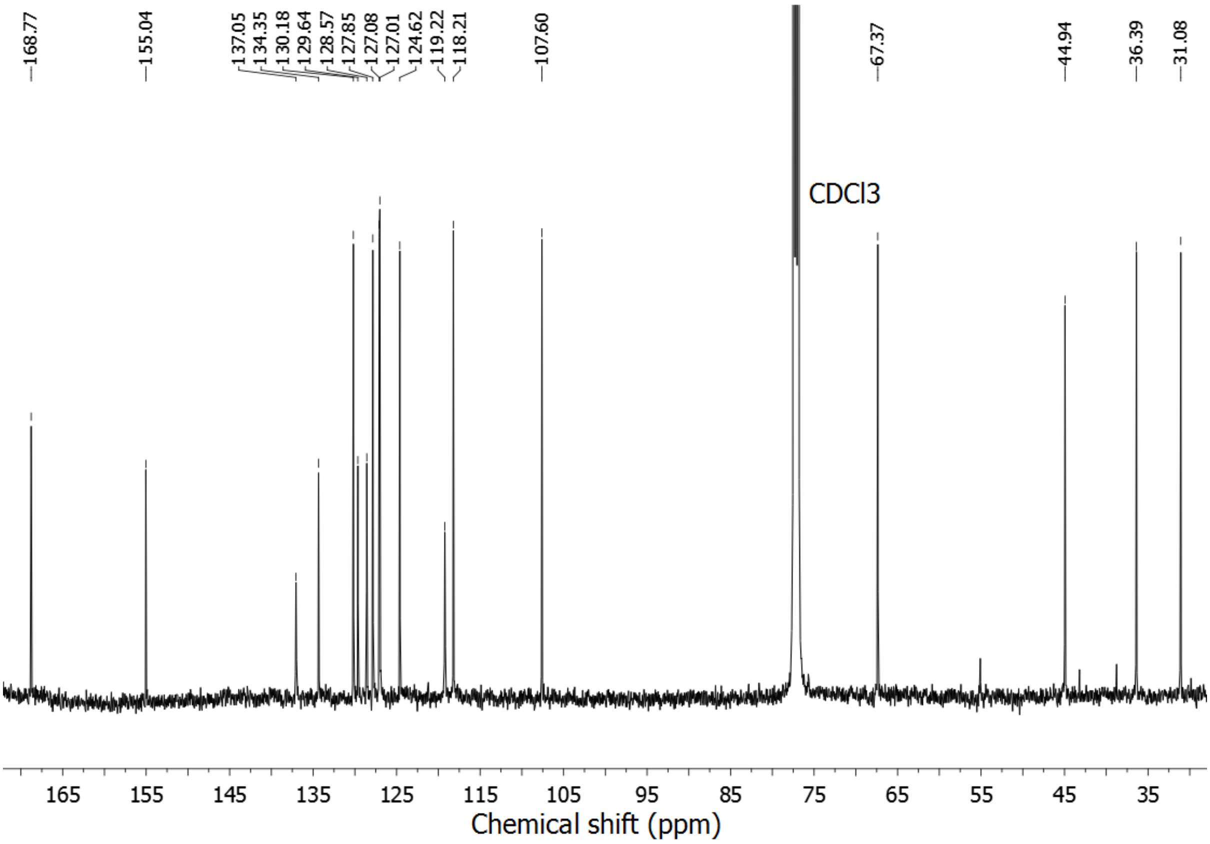

Supplementary Figure 14 | ^{13}C NMR spectrum of G2 (100 MHz, $CDCl_3$, 298 K).

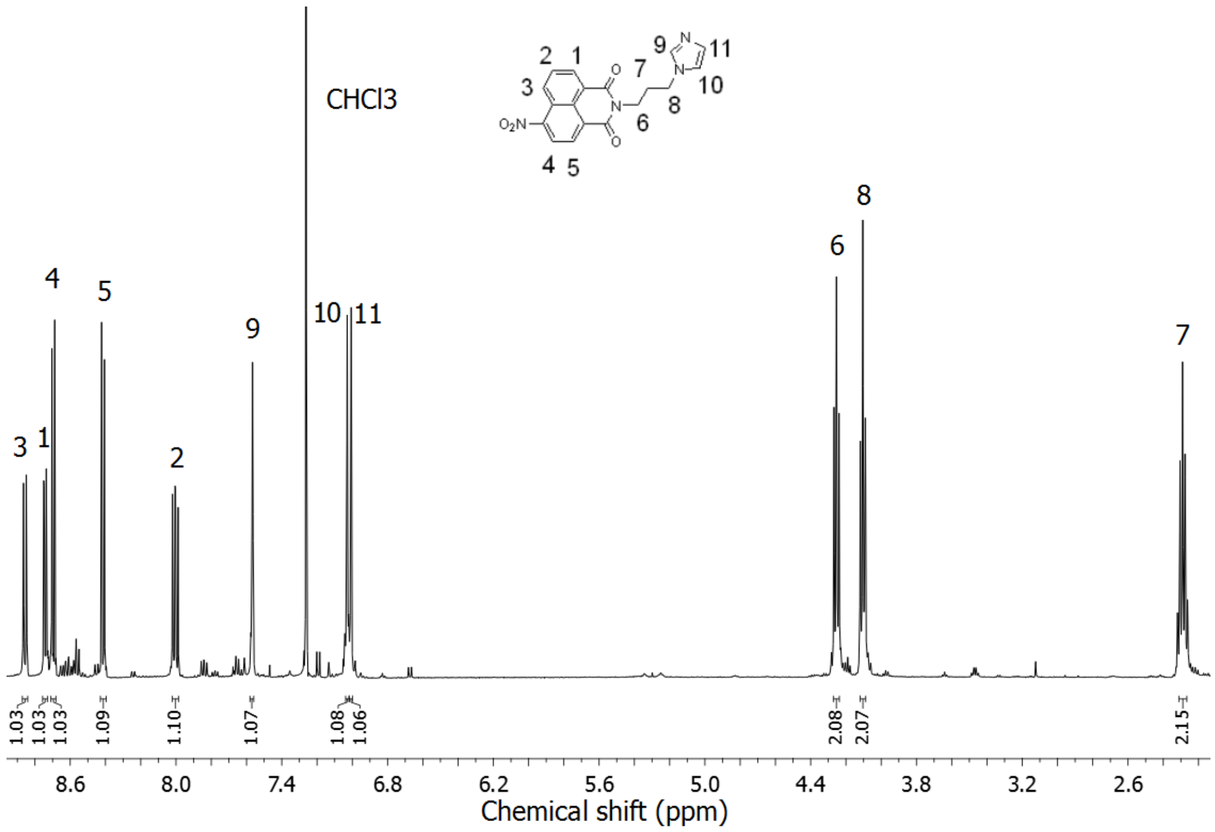

Supplementary Figure 15 | ^{1}H NMR spectrum of G3 (400 MHz, $CDCl_3$, 298 K).

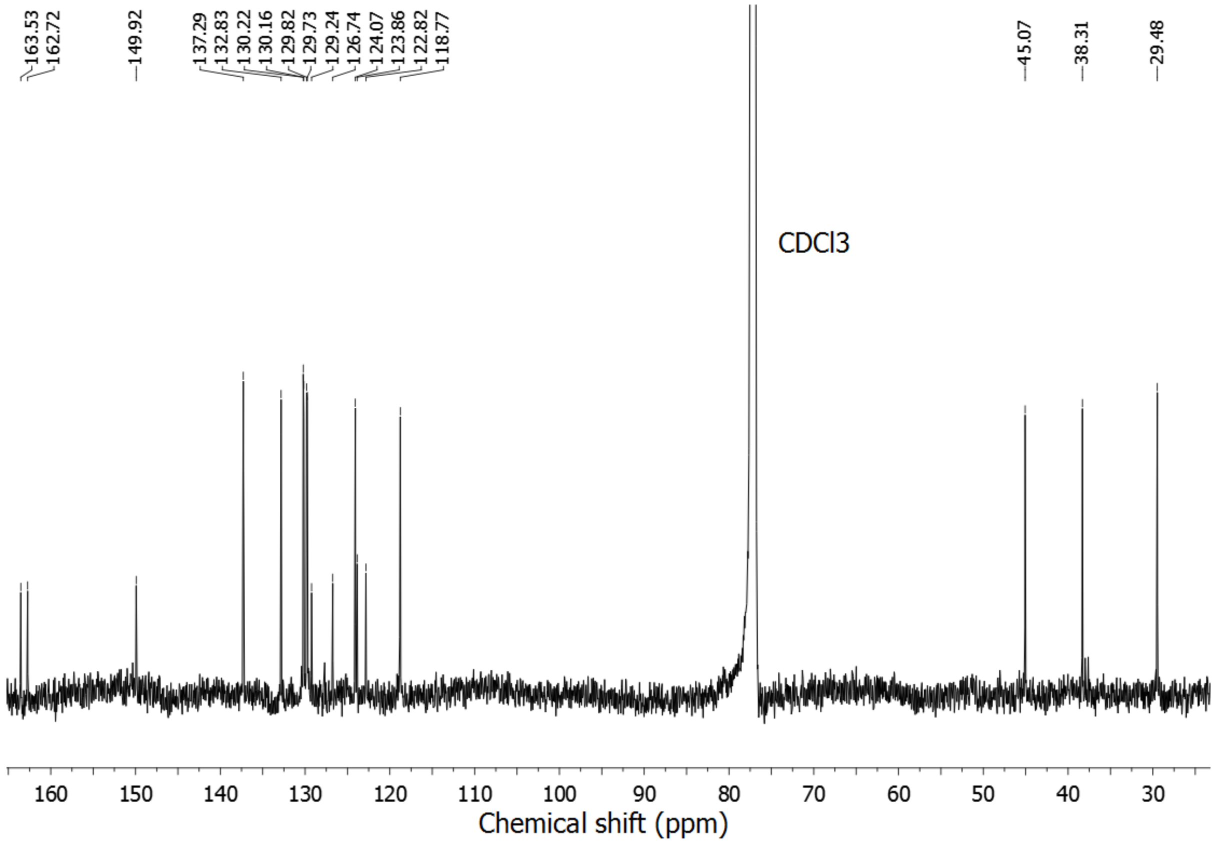

Supplementary Figure 16 |^{13}C NMR spectrum of G3 (100 MHz, $CDCl_3$, 298 K).

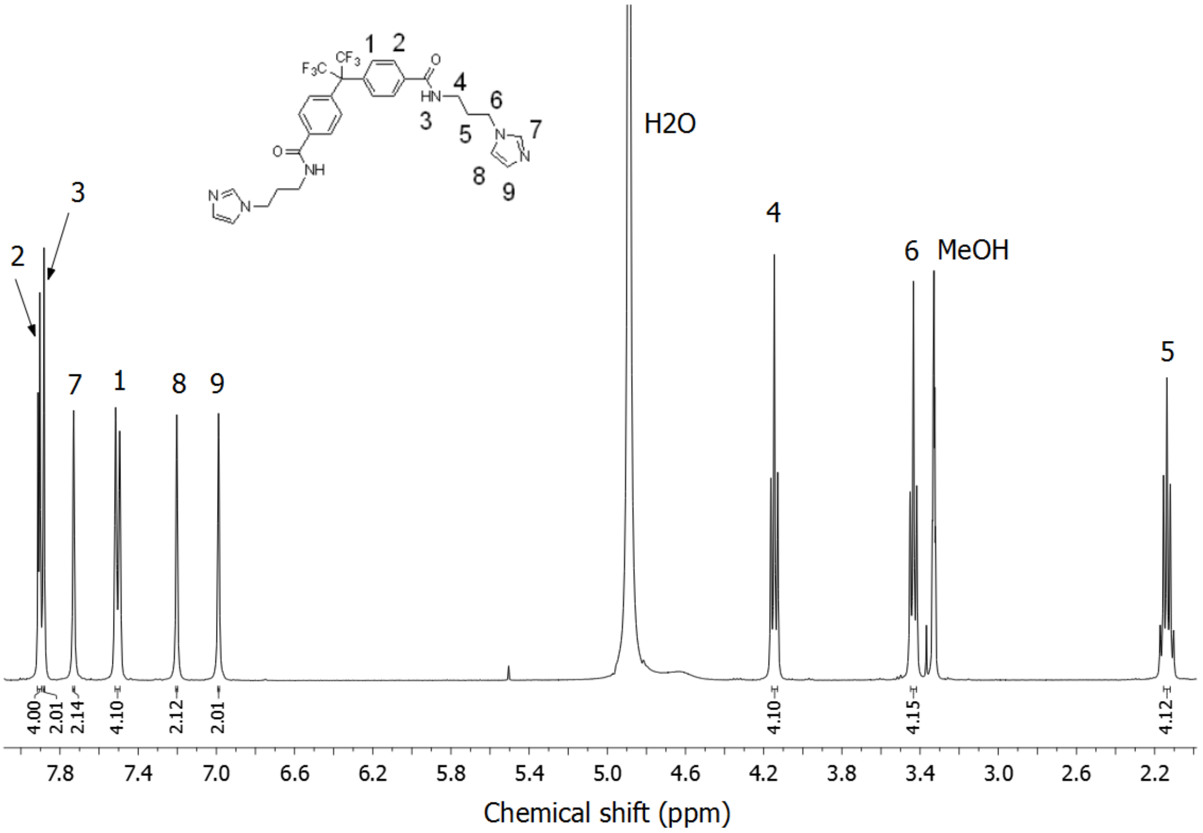

Supplementary Figure 17 | ^{1}H NMR spectrum of G4 (400 MHz, MeOD, 298 K).

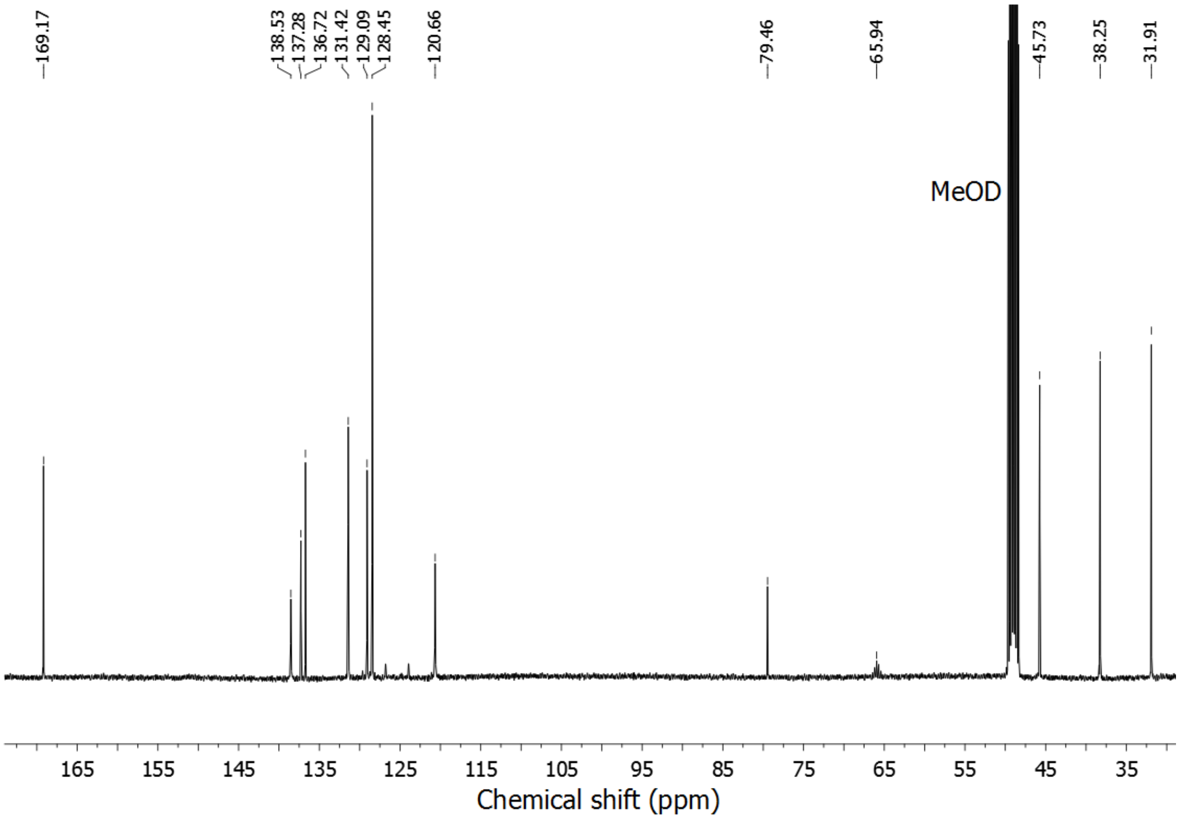

Supplementary Figure 18 | ^{13}C NMR spectrum of G4 (100 MHz, MeOD, 298 K).

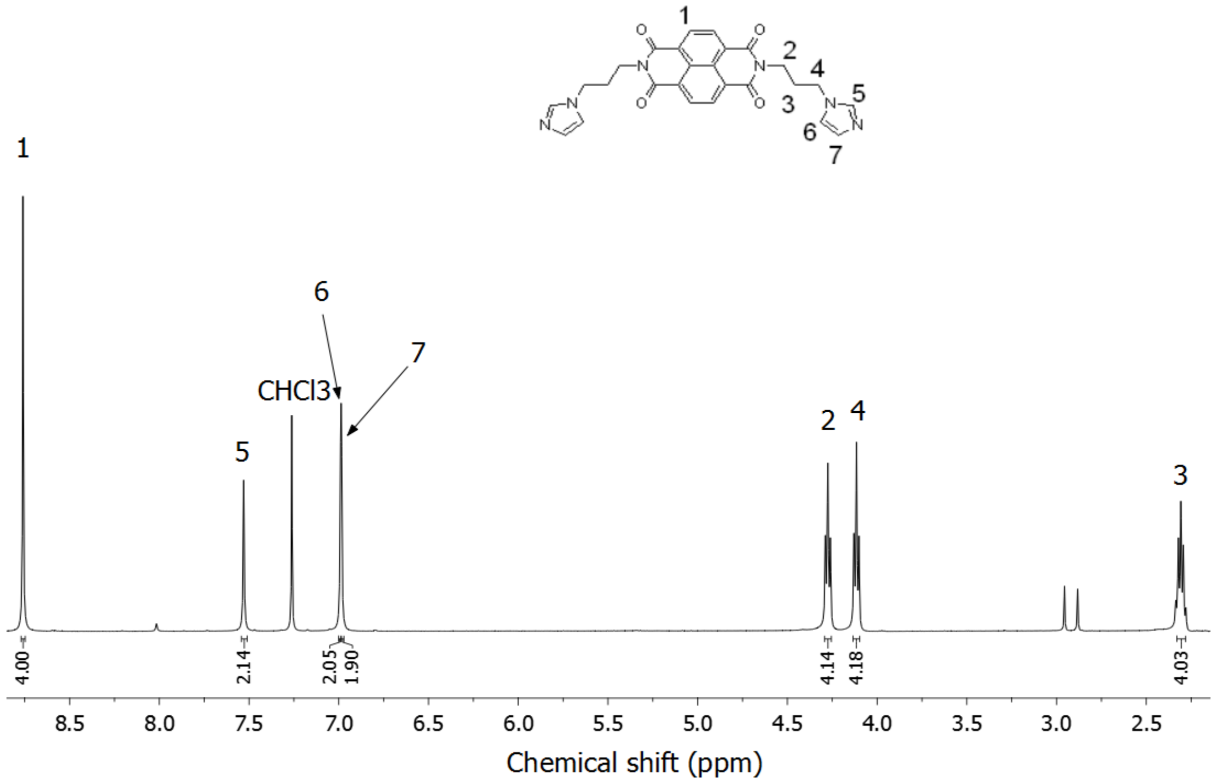

Supplementary Figure 19 | ^{1}H NMR spectrum of G5 (400 MHz, $CDCl_3$, 298 K).

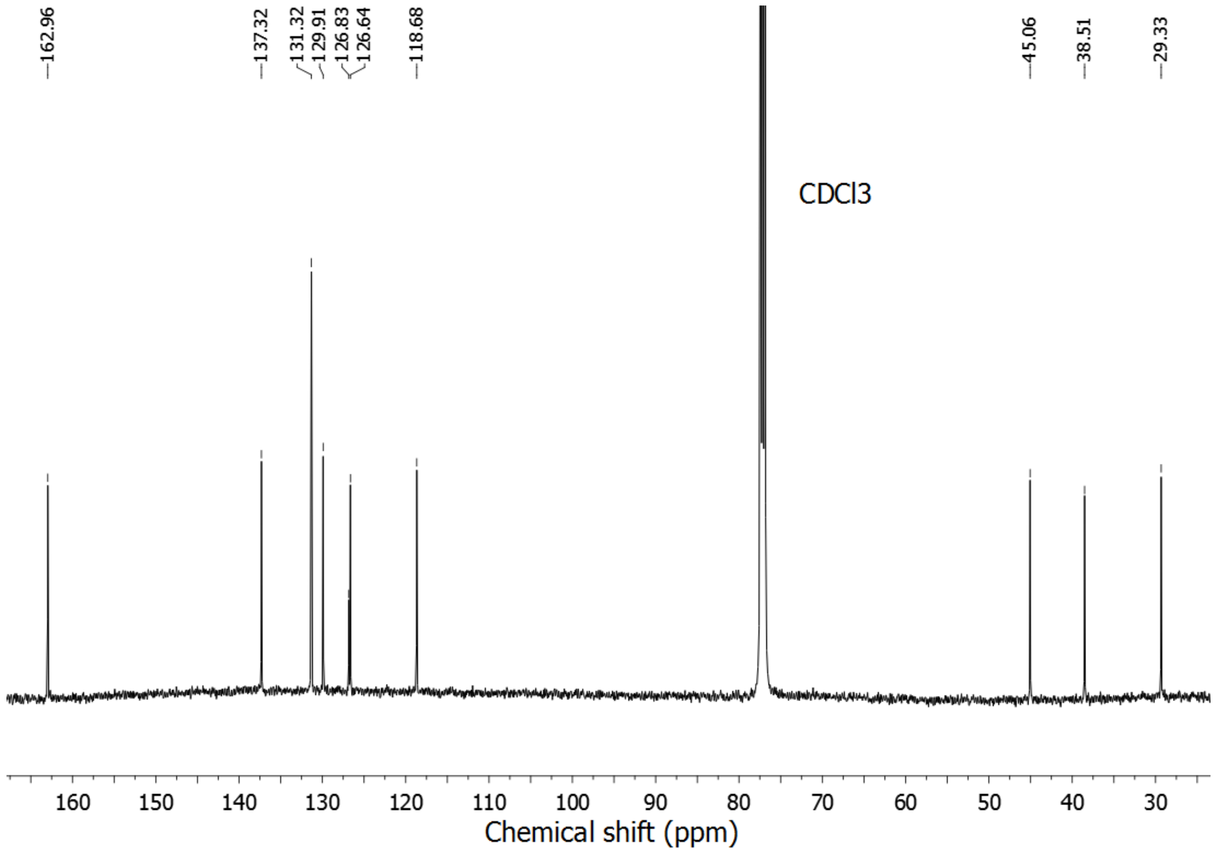

Supplementary Figure 20 | ^{13}C NMR spectrum of G5 (100 MHz, $CDCl_3$, 298 K).

a **b**

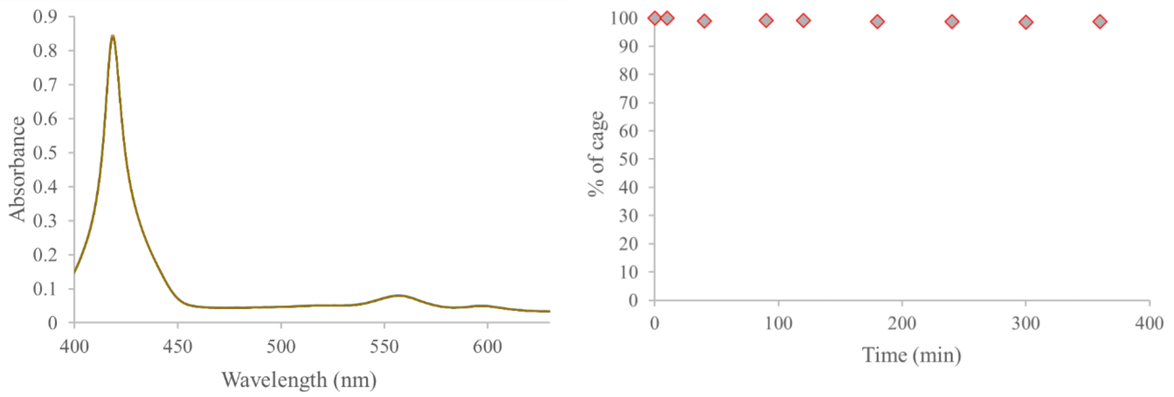

Supplementary Figure 21 | Stability of 1 at 1.5 µM in CH3CN by UV-Vis spectroscopy. (**a**) Variation with the time of the UV-Vis spectrum of the cage **1** (1.5 μM) in CH_3CN at 20 ºC. (**b**) Graphical representation of the decrease of cage concentration with the time due to the degradation based on absorbance measurements (417 nm).

a **b**

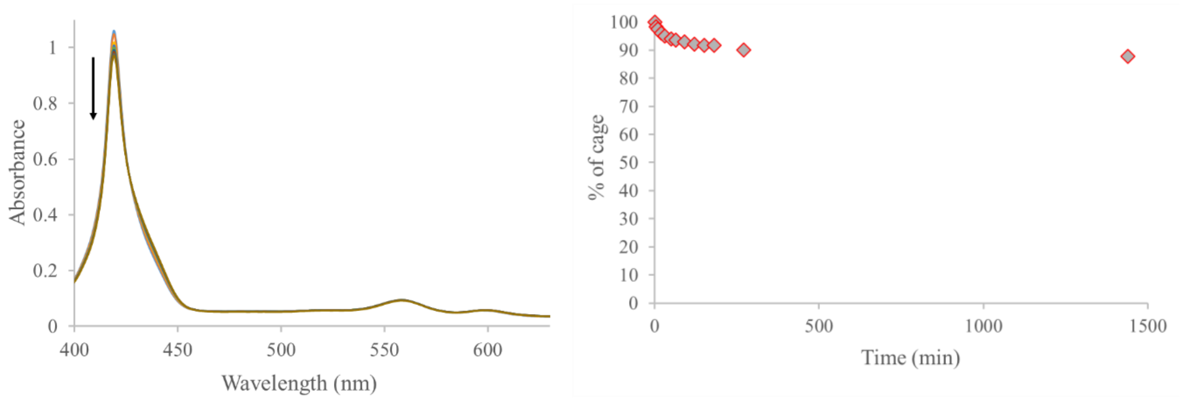

Supplementary Figure 22 | Stability of 1 at 1.5 µM in a mixture 1:1 CH_3CN:H_2O by UV-Vis spectroscopy. (**a**) Variation with the time of the UV-Vis spectrum of the cage **1** (1.5 μM) in a mixture CH_3CN:H_2O (1:1) at 20 ºC. (**b**) Graphical representation of the decrease of cage concentration with the time due to the degradation based on absorbance measurements (417 nm).

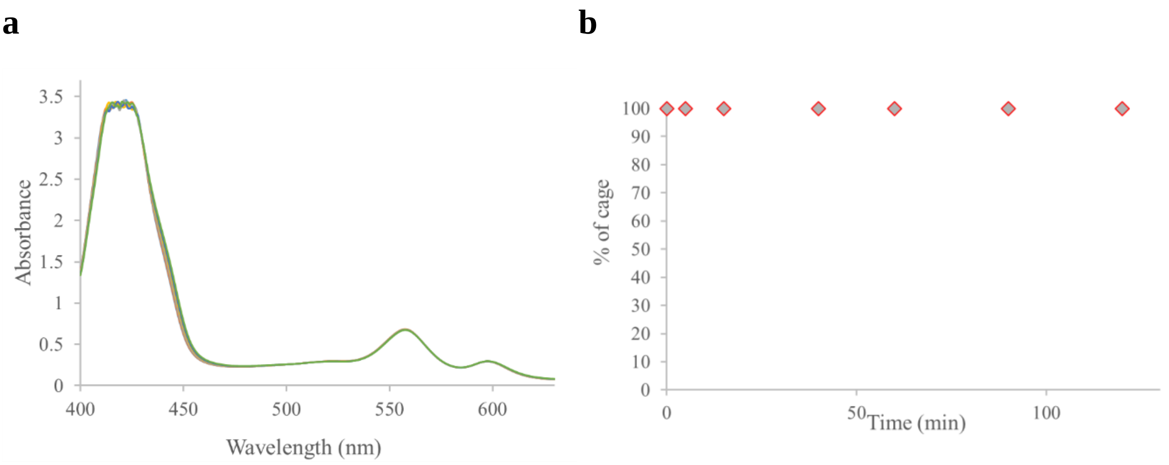

Supplementary Figure 23 | Stability of 1 at 5 µM in a mixture 1:1 $CH_3CN:H_2O$ by UV-Vis spectroscopy. (**a**) Variation with the time of the UV-Vis spectrum of the cage **1** (5 μM) in a mixture $CH_3CN:H_2O$ (1:1) at 20 °C. (**b**) Graphical representation of the decrease of cage concentration with the time due to the degradation based on absorbance measurements (560 nm).

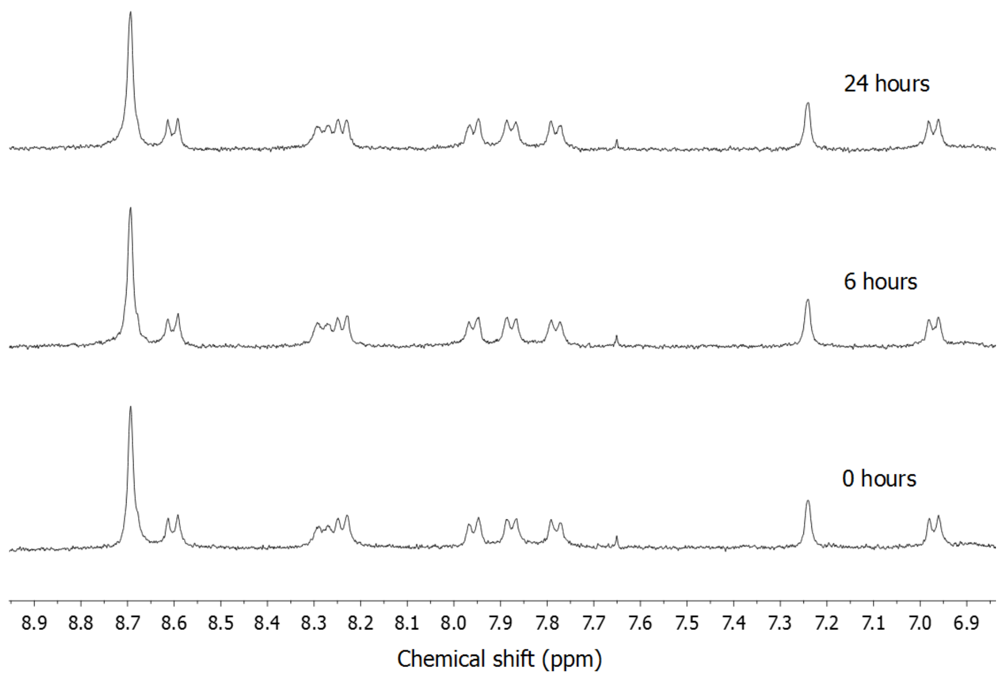

Supplementary Figure 24 | Stability of 1 at 80 µM in a mixture 1:1 $CD_3CN:H_2O$ by ^{1}H NMR. The stability of **1** (80 μM) in a mixture of $CD_3CN:D_2O$ (1:1) monitored by ^{1}H NMR (20°C). ^{1}H NMR spectra of **1** were recorded after 6 and 24 hours. No changes in the spectra were observed after this time.

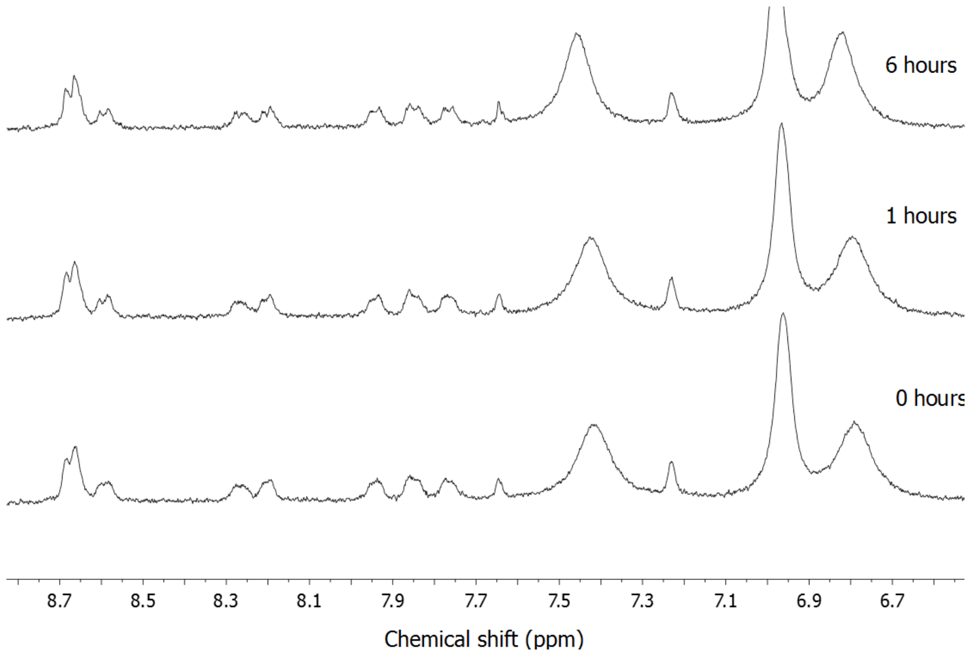

Supplementary Figure 25 | Stability of 1 in the presence of G1 (100 equiv) and in 1:1 CD_3CN:H_2O by ^{1}H NMR. The stability of **1** (50 μM) in the presence of 1-methylimidazole (100 equiv) monitored by ^{1}H NMR (CD_3CN:D_2O (1:1), 20°C). ^{1}H NMR spectra of **1** were recorded after 1 and 6 hours. No changes in the spectra were observed after this time.

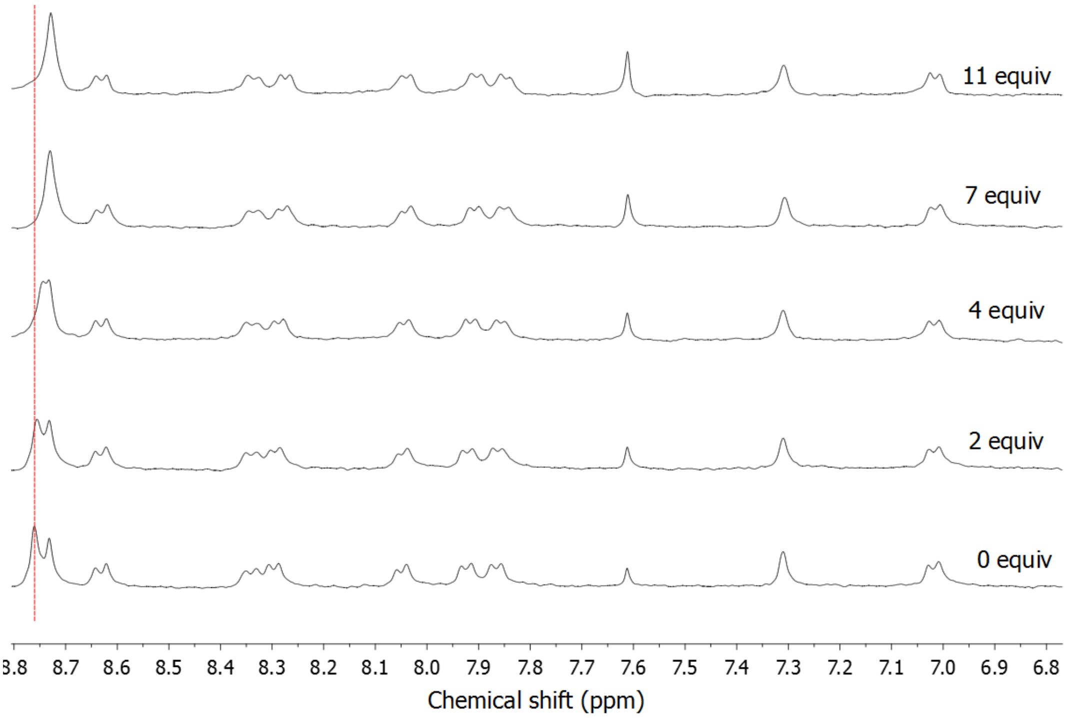

Supplementary Figure 26 | The titration of 1 with G1 (50 μM) as monitored by ^{1}H NMR (400 MHz, CD_3CN, 20°C). Only movements of β-pyrrole signals were observed (red line). Saturation was achieved around 7 equiv of **G1**.

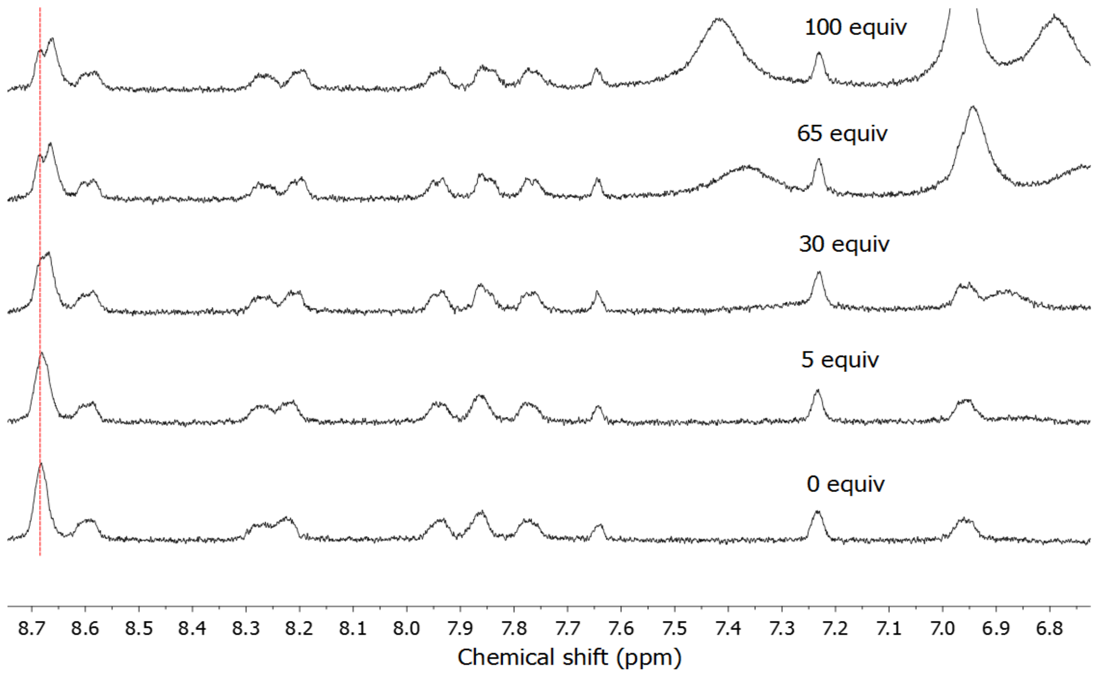

Supplementary Figure 27 | Titration of 1 with G1 (50 μM) as monitored by ^{1}H NMR (400 MHz, CD_3CN:D_2O (1:1), 20°C). Only movements of β-pyrrole signals were observed (red line). The imine and the β-pyrrole peaks are overlap at the beginning.

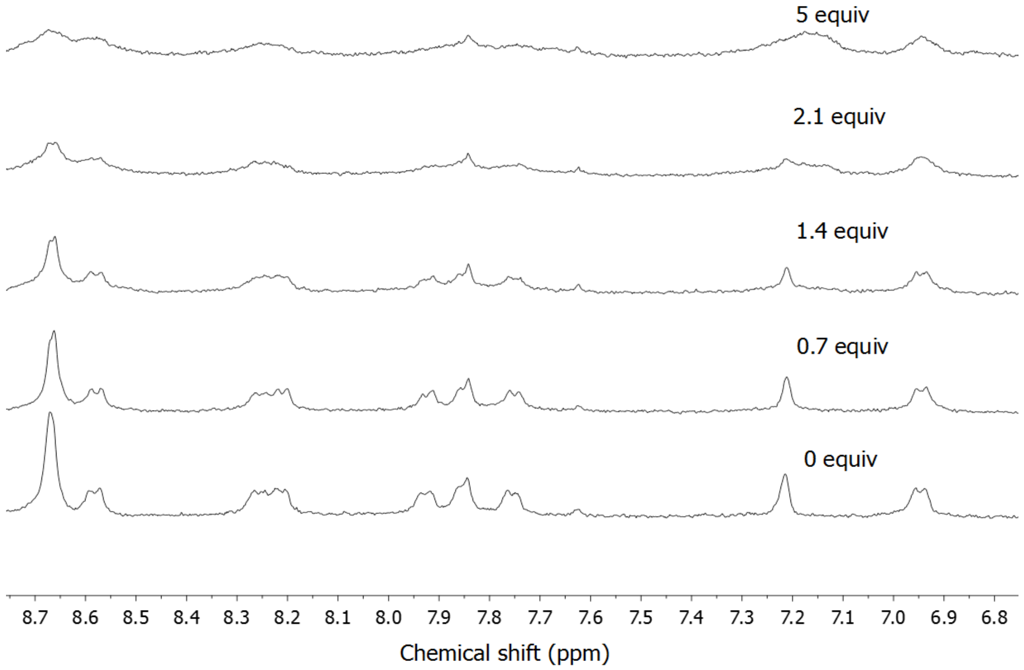

Supplementary Figure 28 | Titration of 1 with G7 (50 μM) as monitored by ^{1}H NMR (400 MHz, CD_3CN:D_2O (1:1), 20°C). The interaction was observed through broadening of the signals due to the desymmeterization caused by the guest binding.

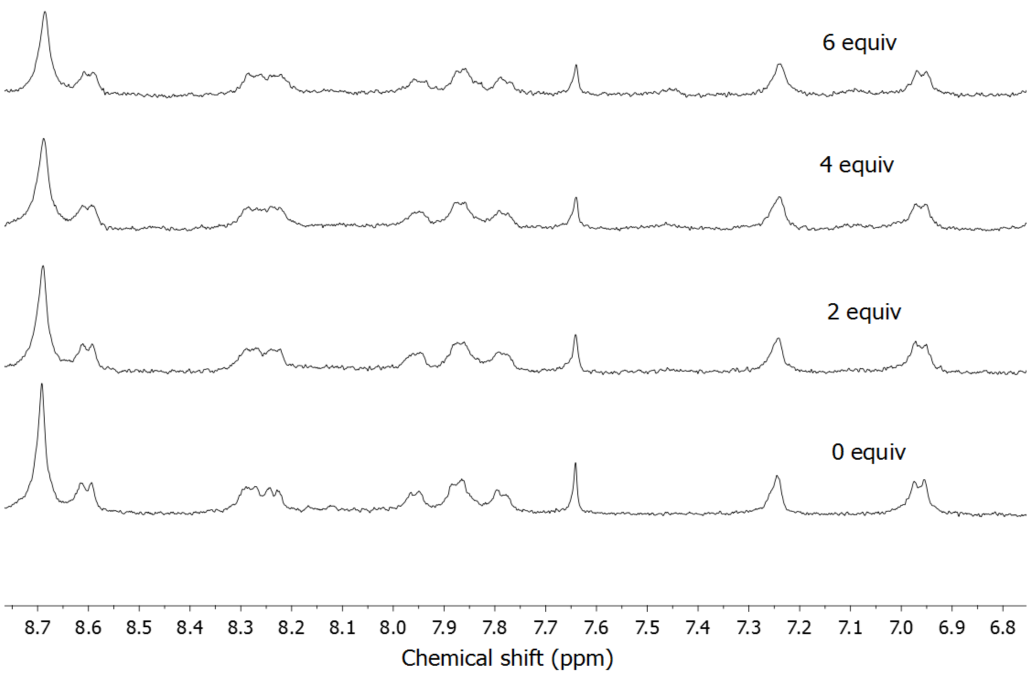

Supplementary Figure 29 | Titration of 1 with G9 (50 μM) as monitored by ^{1}H NMR (400 MHz, CD_3CN:D_2O (1:1), 20°C). No changes were detected after addition of **G9**.

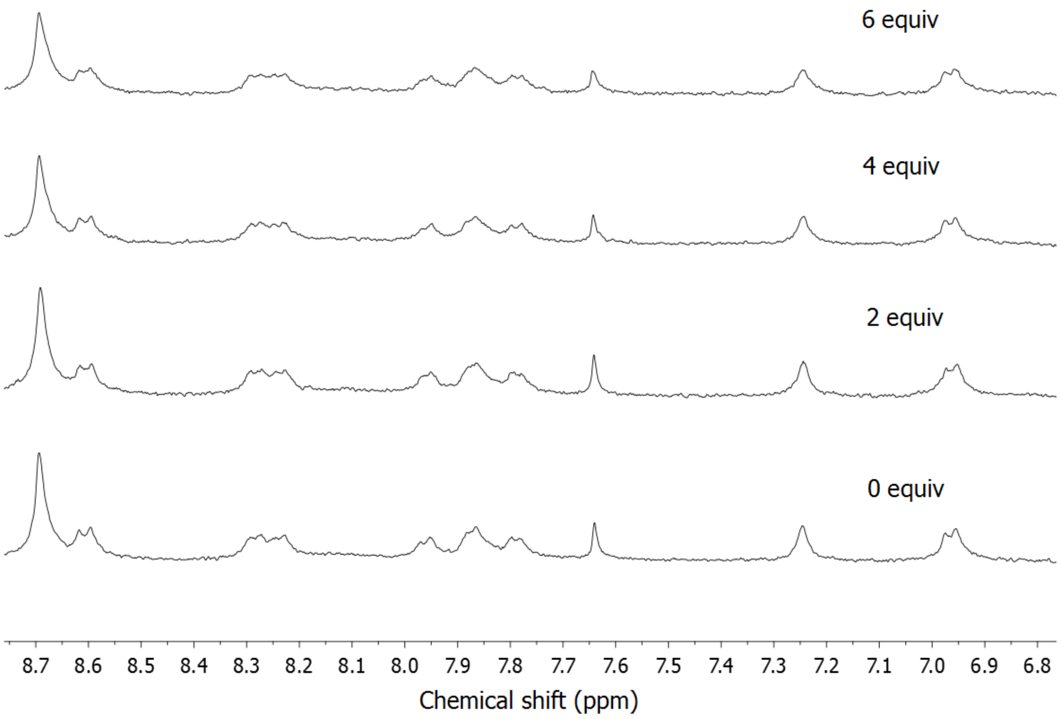

Supplementary Figure 30 | Titration of 1 with G10 (50 μM) as monitored by ^{1}H NMR (400 MHz, CD_3CN:D_2O (1:1), 20°C). No changes were observed after addition of **G10**.

a b

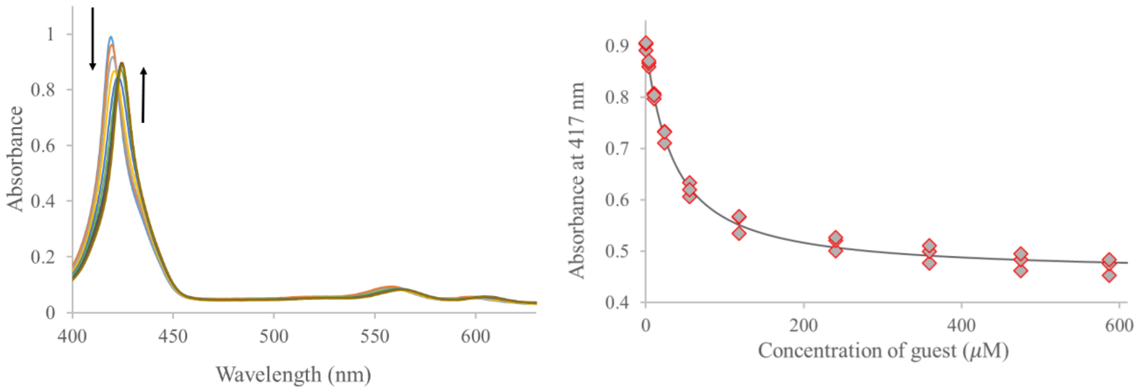

Supplementary Figure 31 | UV-Vis titration of G1 in CH_3CN. (**a**) Titration of **1** with **G1** as monitored by UV-Vis (CH_3CN, 20℃). (**b**) Binding isotherm from UV-Vis analysis (Kd = 30 ± 1 μM at 20℃).

a b

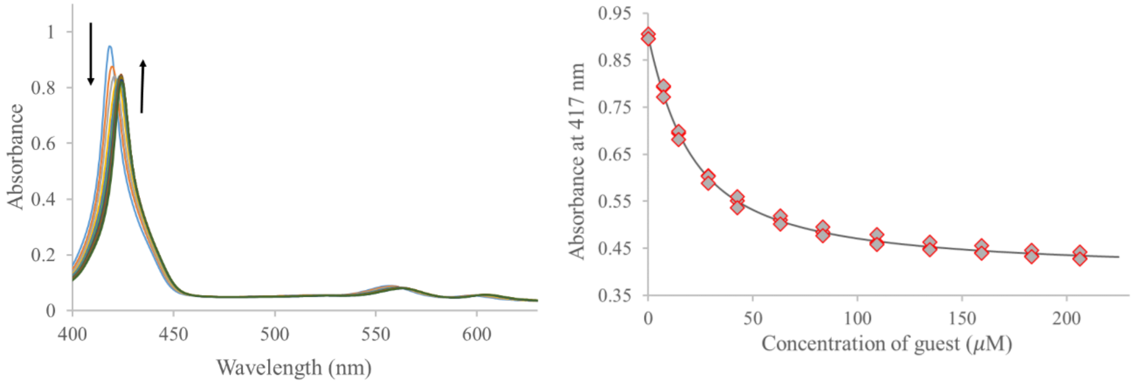

Supplementary Figure 32 | UV-Vis titration of G2 in CH_3CN. (**a**) Titration of **1** with **G2** as monitored by UV-Vis (CH_3CN, 20℃). (**b**) Binding isotherm from UV-Vis analysis (Kd = 15.8 ± 0.7 μM at 20℃).

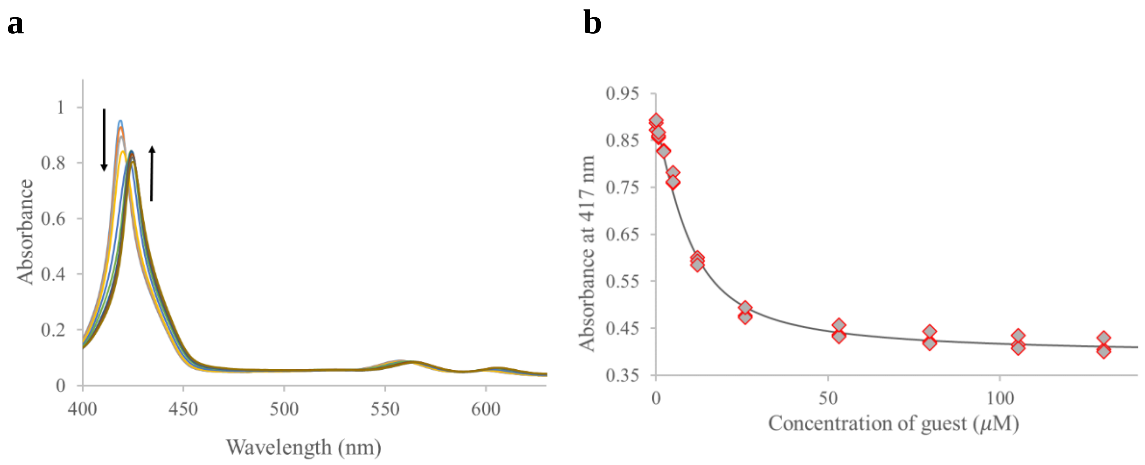

Supplementary Figure 33 | UV-Vis titration of G3 in CH_3CN. (**a**) Titration of **1** with **G3** as monitored by UV-Vis (CH_3CN, 20℃). (**b**) Binding isotherm from UV-Vis analysis (Kd = 5 ± 0.5 μM at 20℃).

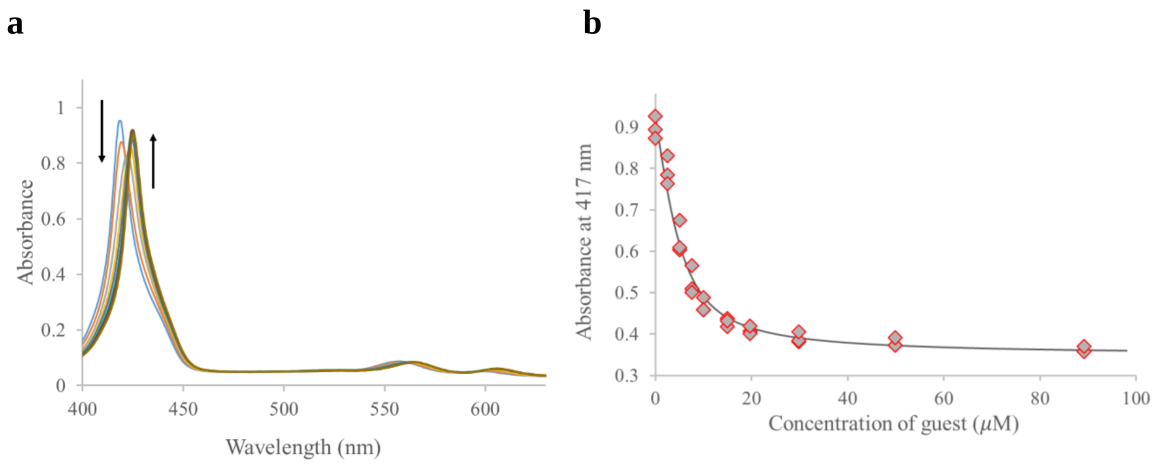

Supplementary Figure 34 | UV-Vis titration of G4 in CH_3CN. (**a**) Titration of **1** with **G4** as monitored by UV-Vis (CH_3CN, 20℃). (**b**) Binding isotherm from UV-Vis analysis (Kd = 2.0 ± 0.3 μM at 20℃).

a **b**

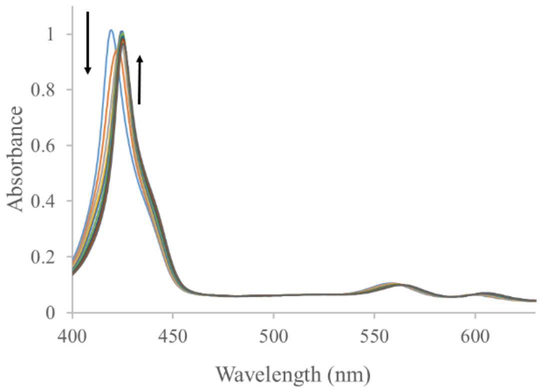

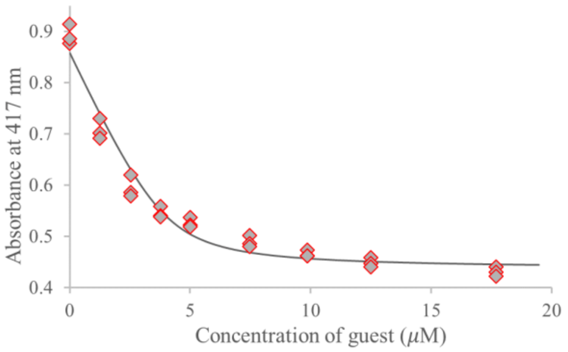

Supplementary Figure 35 | UV-Vis titration of G5 in CH_3CN. (**a**) Titration of **1** with **G5** as monitored by UV-Vis (CH_3CN, 20°C). (**b**) Binding isotherm from UV-Vis analysis (*Kd* = 0.3 ± 0.1 μM at 20°C).

a **b**

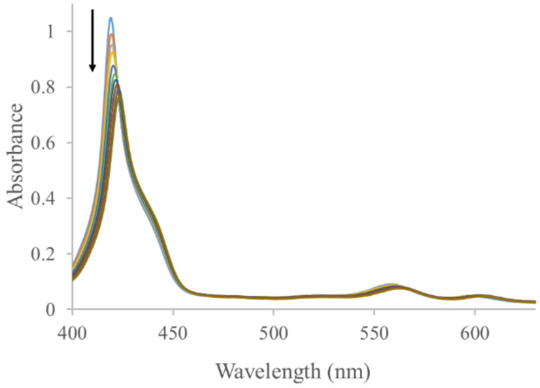

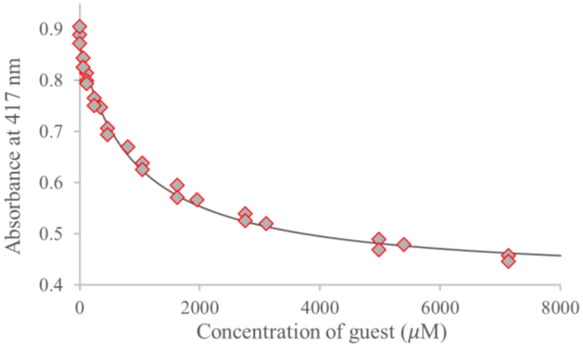

Supplementary Figure 36 | UV-Vis titration of G1 in CH_3CN:H_2O (1:1). (**a**) Titration of **1** with **G1** as monitored by UV-Vis (CH_3CN:H_2O, 20°C). (**b**) Binding isotherm from UV-Vis analysis (*Kd* = 900 ± 70 μM at 20°C).

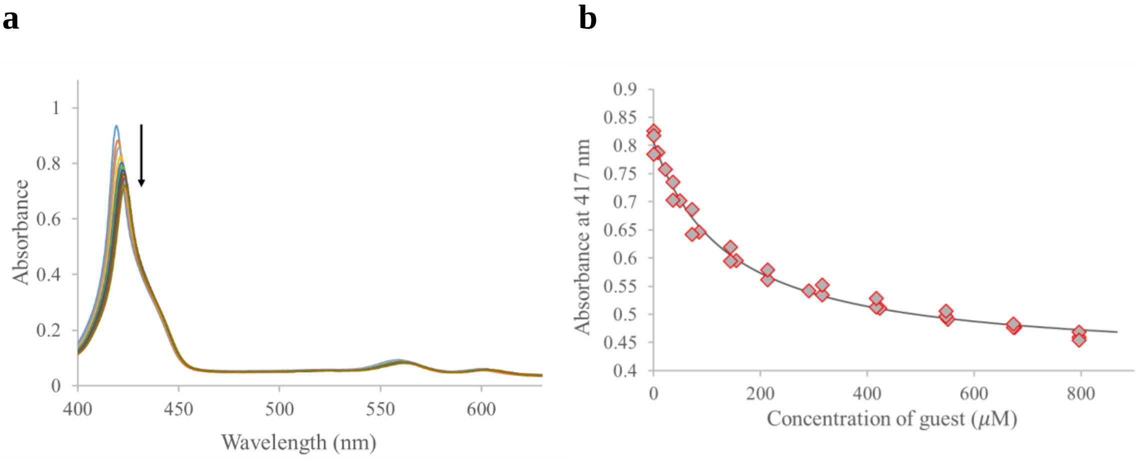

Supplementary Figure 37 | UV-Vis titration of G2 in CH_3CN:H_2O (1:1). (**a**) Titration of **1** with **G2** as monitored by UV-Vis (CH_3CN:H_2O, 20°C). (**b**) Binding isotherm from UV-Vis analysis (Kd = 130 ± 10 μM at 20°C).

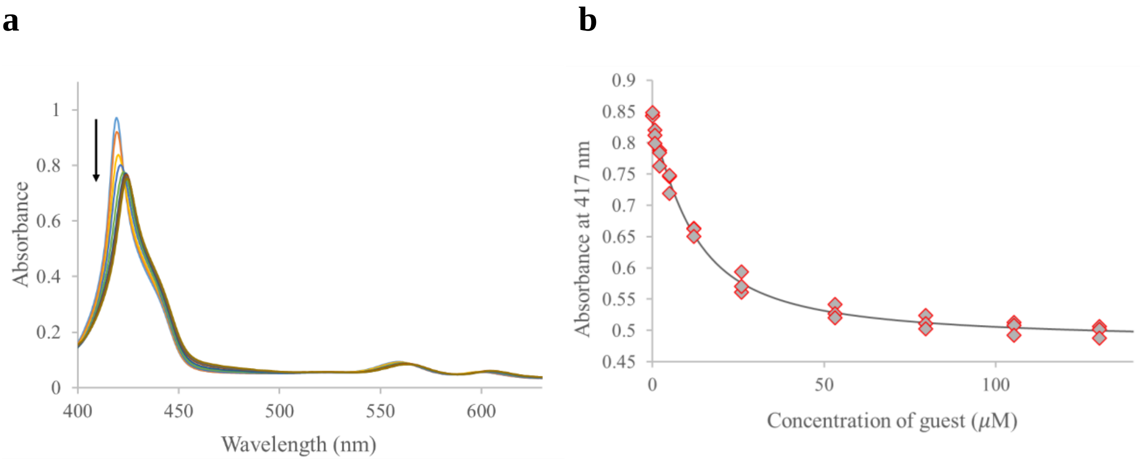

Supplementary Figure 38 | UV-Vis titration of G3 in CH_3CN:H_2O (1:1). (**a**) Titration of **1** with **G3** as monitored by UV-Vis (CH_3CN:H_2O, 20°C). (**b**) Binding isotherm from UV-Vis analysis (Kd = 7.3 ± 0.8 μM at 20°C).

a **b**

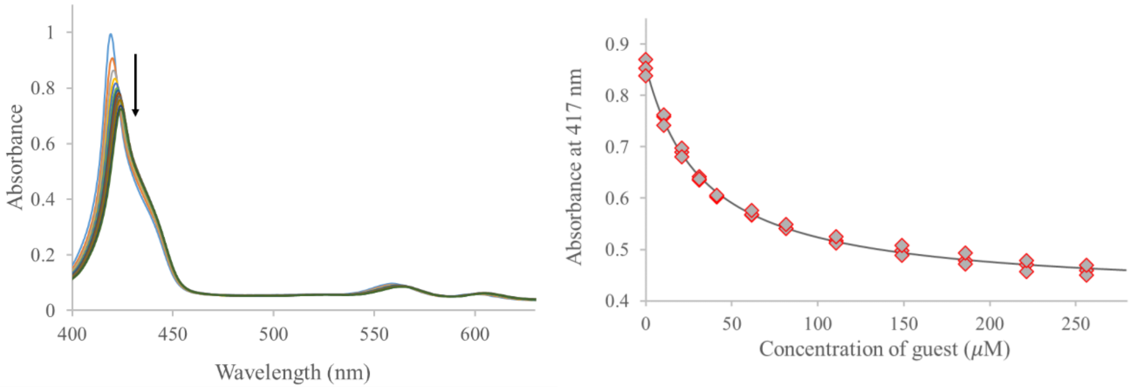

Supplementary Figure 39 | UV-Vis titration of G4 in CH_3CN:H_2O (1:1). (**a**) Titration of **1** with **G4** as monitored by UV-Vis (CH_3CN:H_2O, 20℃). (**b**) Binding isotherm from UV-Vis analysis (Kd = 31 ± 2 μM at 20℃).

a **b**

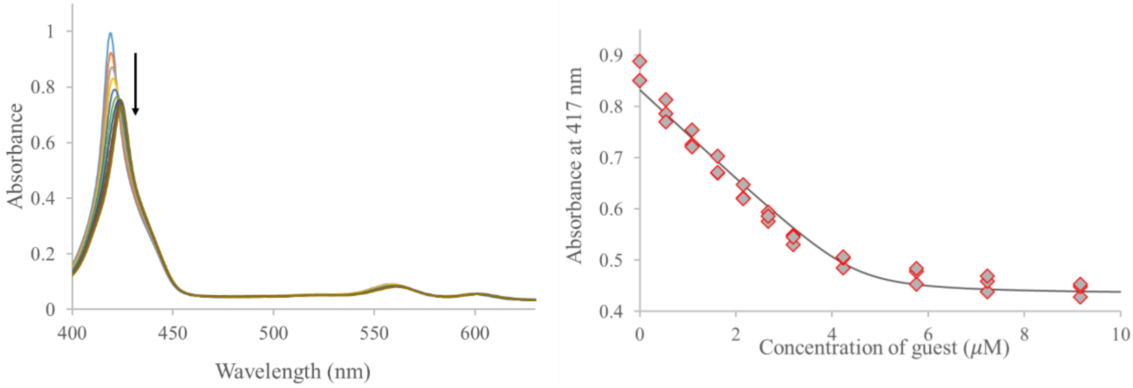

Supplementary Figure 40 | UV-Vis titration of G5 in CH_3CN:H_2O (1:1). (**a**) Titration of **1** with **G5** as monitored by UV-Vis (CH_3CN:H_2O, 20℃). (**b**) Binding isotherm from UV-Vis analysis (Kd = 0.08± 0.06 μM at 20℃).

a **b**

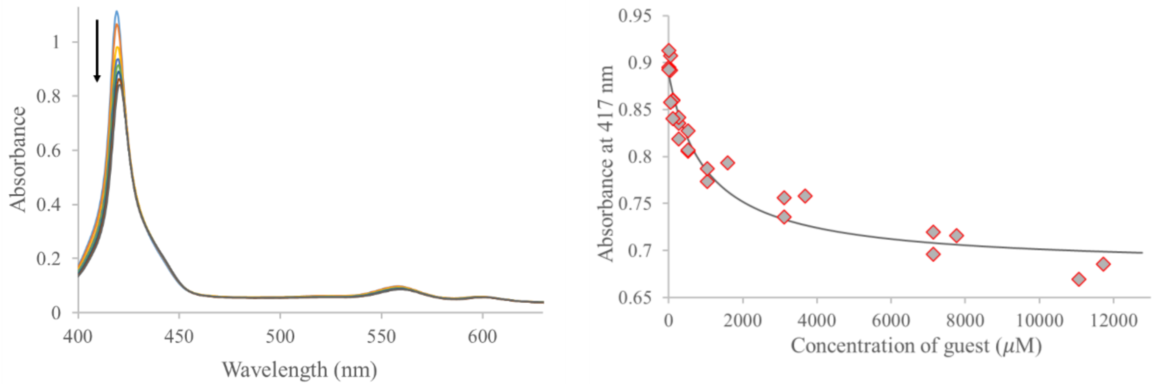

Supplementary Figure 41 | UV-Vis titration of 1 with thiazole in CH_3CN:H_2O (1:1). (**a**) Titration of **thiazole** with **1** as monitored by UV-Vis (CH_3CN:H_2O, 20℃). (**b**) Binding isotherm from UV-Vis analysis (Kd = 1000 ± 200 μM at 20℃).

a **b**

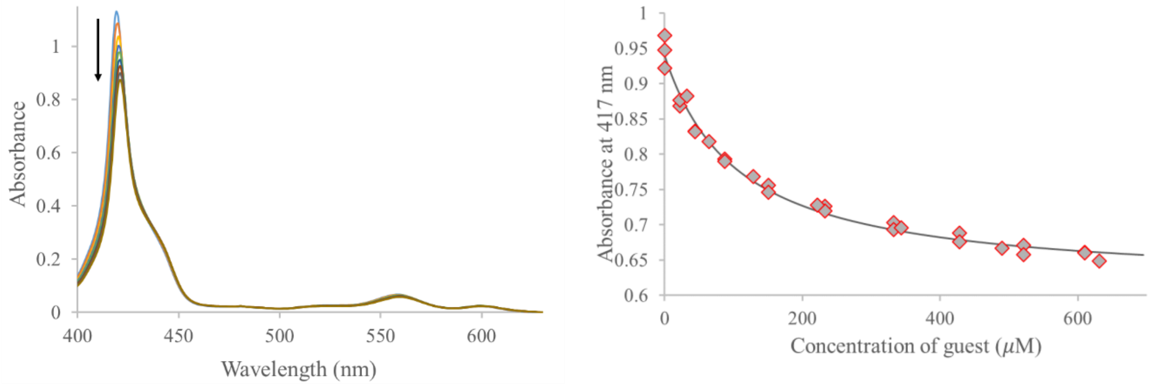

Supplementary Figure 42 | UV-Vis titration of G6 in CH_3CN:H_2O (1:1). (**a**) Titration of **1** with **G6** as monitored by UV-Vis (CH_3CN:H_2O, 20℃). (**b**) Binding isotherm from UV-Vis analysis (Kd = 107 ± 10 μM at 20℃).

a **b**

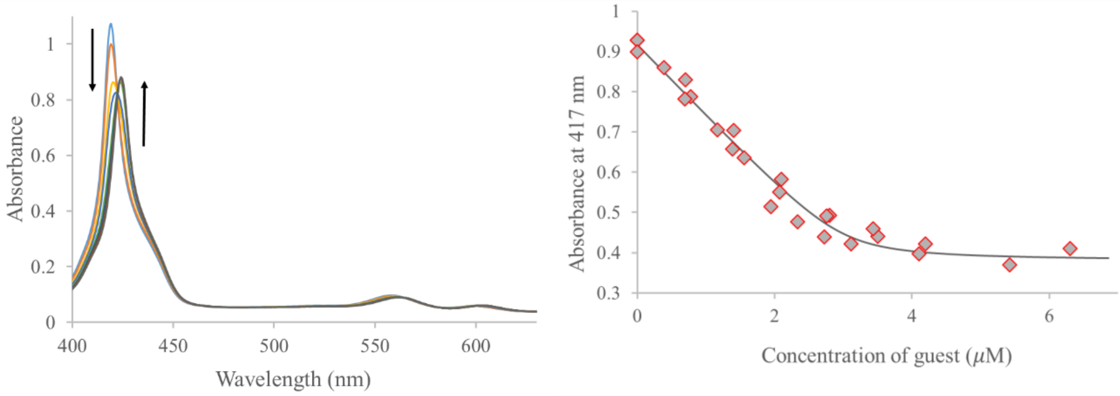

Supplementary Figure 43 | UV-Vis titration of G7 in CH_3CN:H_2O (1:1). (**a**) Titration of **1** with **G7** as monitored by UV-Vis (CH_3CN:H_2O, 20°C). (**b**) Binding isotherm from UV-Vis analysis. (*Kd* = 60 ± 50 nM at 20°C). This titration proved that the cage is saturated with only two equivalents of **G7** (3μM).

a **b**

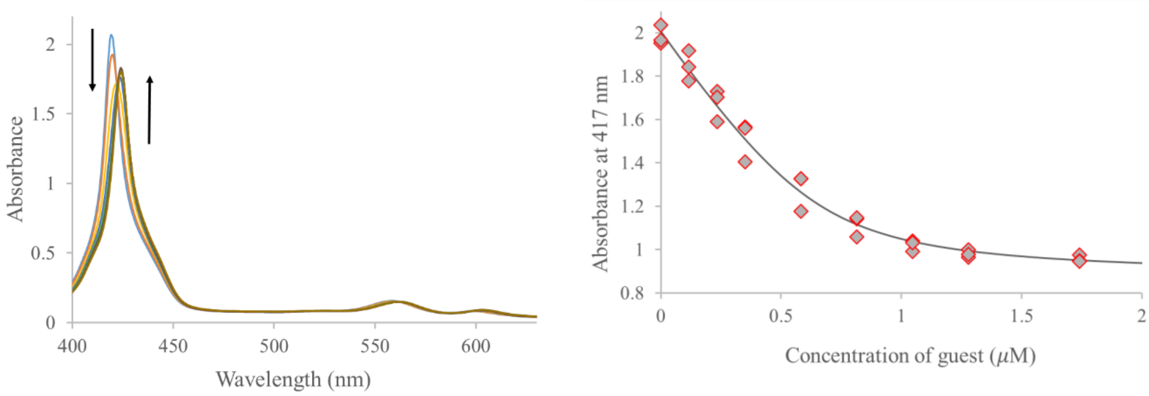

Supplementary Figure 44 | UV-Vis titration of G7 in CH_3CN:H_2O (1:1). (**a**) Titration of **1** with **G7** as monitored by UV-Vis (CH_3CN:H_2O, 20°C). (**b**) Binding isotherm from UV-Vis analysis (*Kd* = 80 ± 20 nM at 20°C). The titration showed in Supplementary Figure 43 was repeated here under more dilute conditions (300 nM) to obtain a more accurate value for the Kd. Although the degradation is faster at this concentration, no significance reduction of the signal was observed over the duration of the experiment. The reduction of the signal at 417 nm is similar to the result obtain when the experiment was accomplished at 1.5 μM. This experiment was carried out using quartz cuvettes with optical path lengths of 10 mm.

a **b**

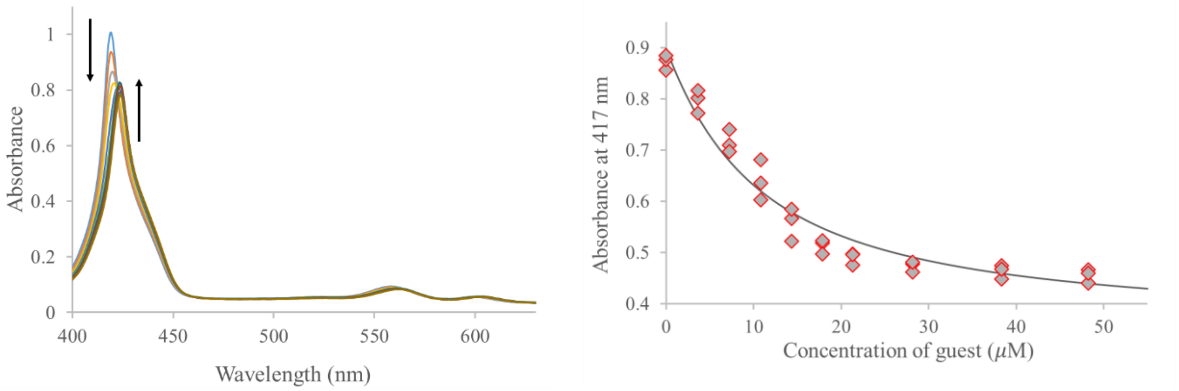

Supplementary Figure 45 | UV-Vis titration of G8 in CH_3CN:H_2O (1:1). (**a**) Titration of **1** with **G8** as monitored by UV-Vis (CH_3CN:H_2O, 20℃). (**b**) Binding isotherm from UV-Vis analysis (Kd = 9 ± 2 μM at 20℃).

a **b**

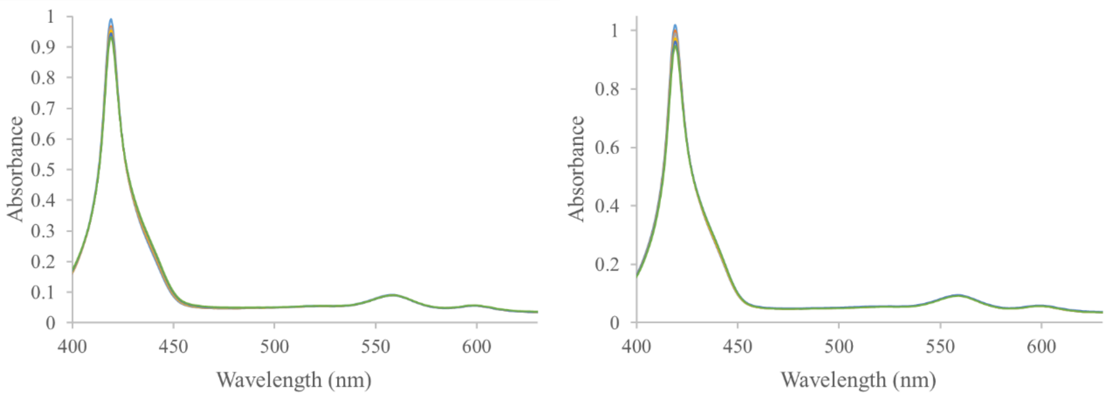

Supplementary Figure 46 | UV-Vis titration of G9 and G10 in CH_3CN:H_2O (1:1)**.** (**a**) Titration of **1** with **G9** (1, 2, 4, 8 and 12 equiv) as monitored by UV-Vis (CH_3CN: H_2O (1:1), 20℃). (**b**) Titration of **1** with **G10** (1, 2, 4, 8 and 12 equiv) as monitored by UV-Vis (CH_3CN: H_2O (1:1), 20℃). The results showed that there are no binding interaction between **1** and the guest in both cases

a **b**

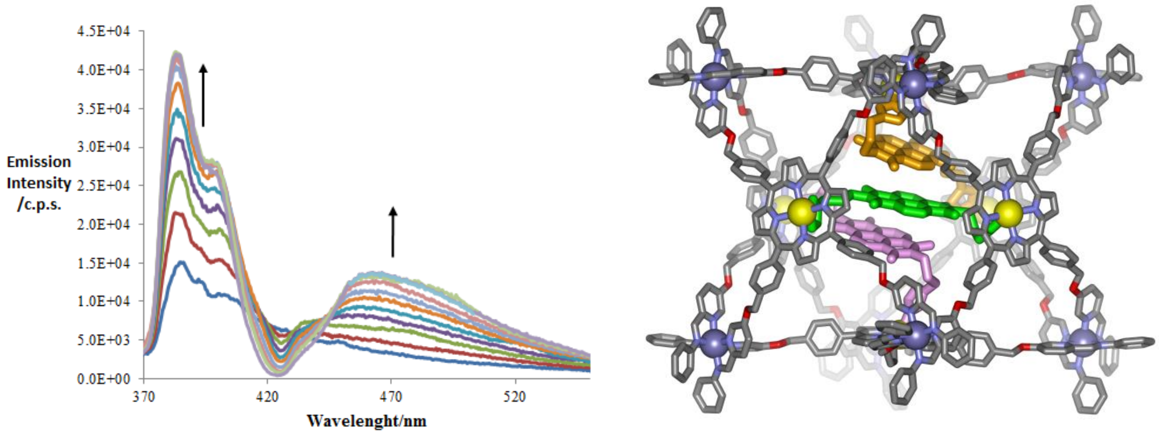

Supplementary Figure 47 | G5 interaction with 1. (**a**) Fluorescence spectra recorded during the titration of **G5** with **1** in a 1:1 water/acetonitrile solution. (**b**) MM2-optimized molecular model of the cage **1** interacting with three molecules of **G5**.

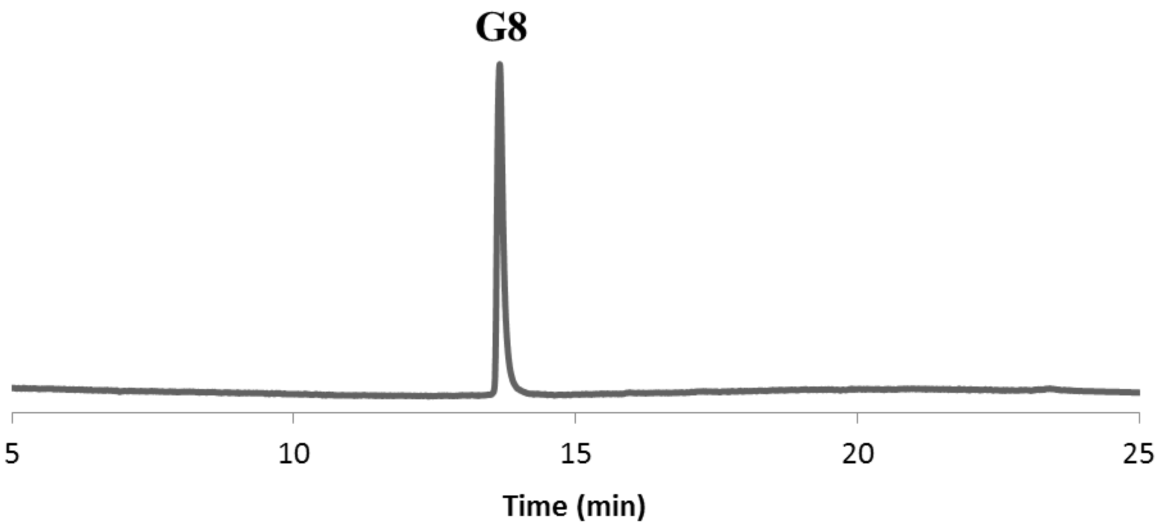

Supplementary Figure 48 | G8 studied by HPLC. **G8** was observed at 13.7 minutes. ESI-MS of **G8:** calcd. for $[C_{88}H_{122}N_{26}O_{23}]^{+2}$ = 956.0, found = 956.3.

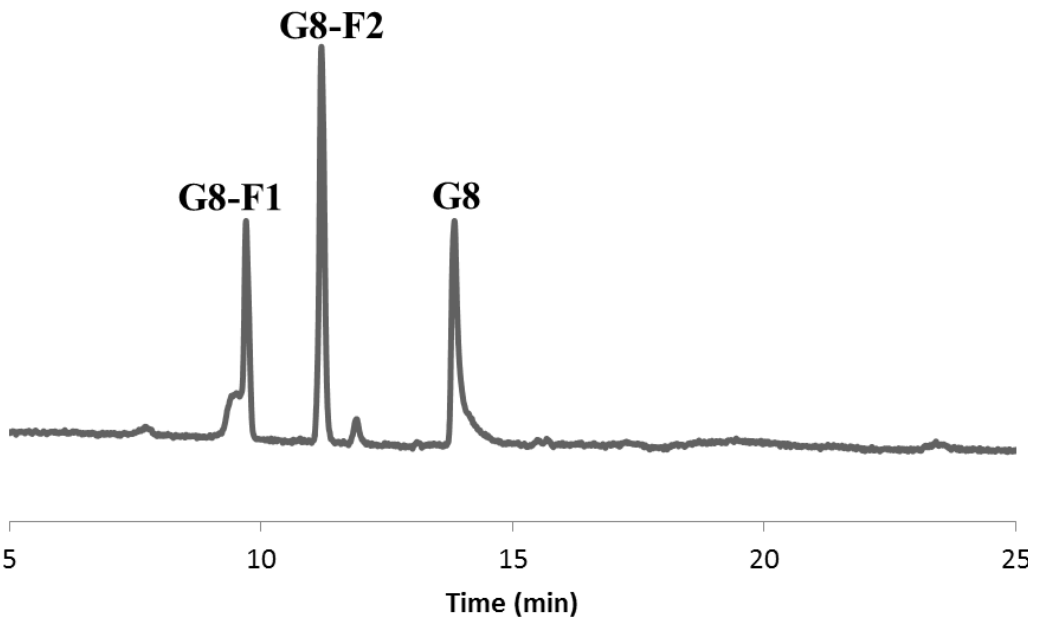

Supplementary Figure 49 | Trypsinization of G8 studied by HPLC. **G8–F1** (N-terminal fragment) and **G8-F2** (C-terminal fragment) are peptide fragments of **G8**. **G8–F1**, **G8-F2** and **G8** were observed at 9.7, 11.1, and 13.7 minutes respectively. 76% of peptide **G8** was cleaved by the enzyme. ESI-MS of **G8-F1:** calcd. for $[C_{38}H_{57}N_{12}O_{11}]^+$ = 857.9, found = 857.3. ESI-MS of **G8-F2:** calcd. for $[C_{50}H_{67}N_{14}O_{13}]^+$ = 1072.2, found = 1072.1.

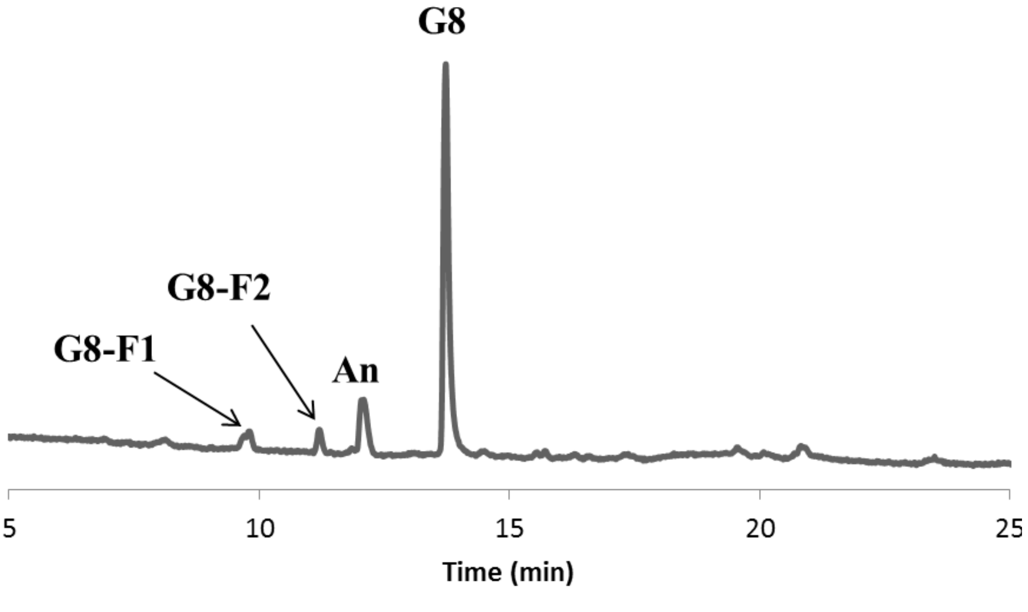

Supplementary Figure 50 | Trypsinization of G8 in the presence of 1 studied by HPLC. **G8–F1** (N-terminal fragment) and **G8-F2** (C-terminal fragment) are peptide fragments of **G8**. **G8–F1**, **G8-F2** and **G8** were observed at 9.7, 11.1, and 13.7 minutes respectively. **An** is 5,6,7,8-tetrahydronaphthalen-2-amine from **1**. Only 9 % of peptide **G8** was cleaved by the enzyme.

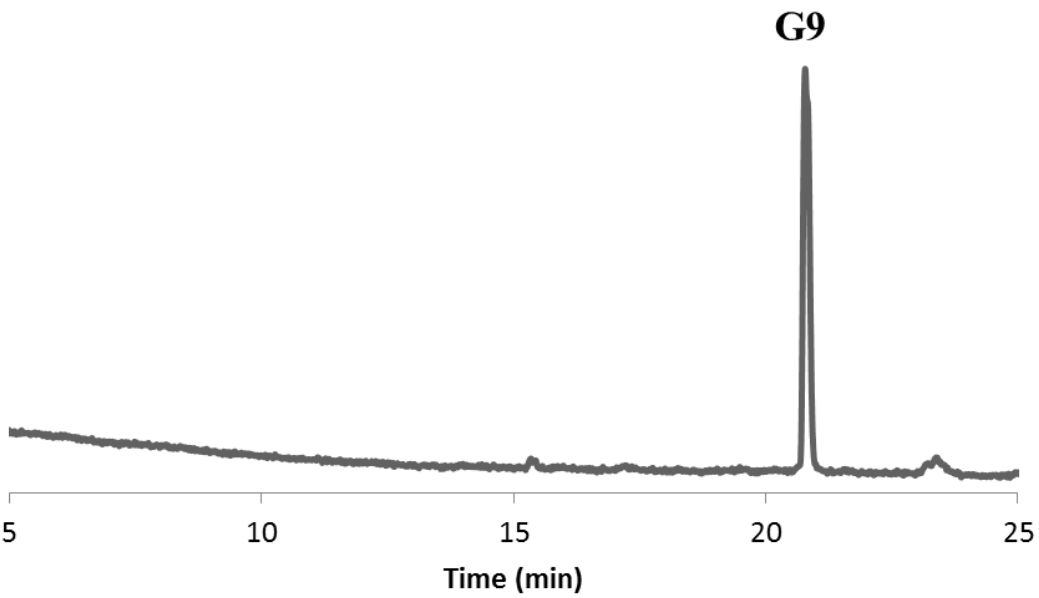

Supplementary Figure 51 | G9 studied by HPLC. **G9** was observed at 20.8 minutes. ESI-MS of **G9:** calcd. for $[C_{41}H_{62}N_{13}O_{12}]^+$ = 929.0 found = 928.7.

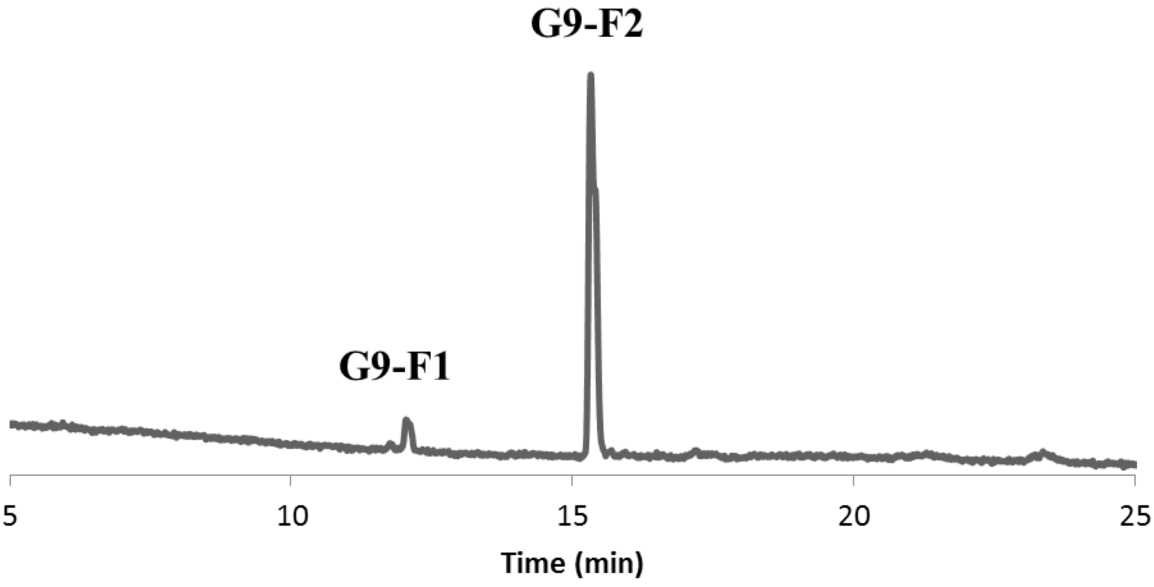

Supplementary Figure 52 | Trypsinization of G9 studied by HPLC. **G9-F1** (N-terminal) and **G9-F2** (C-terminal) are peptide fragments of **G9**. **G9-F1** and **G9-F2** were observed at 12.0 and 15.3 minutes respectively. 100% of peptide **G9** was cleaved. ESI-MS of **G9-F1:** calcd. for $[C_{26}H_{43}N_8O_6]^+$ = 563.7, found = 563.7. ESI-MS of **G9-F2:** calcd. for $[C_{15}H_{22}N_5O_7]^+$ = 384.4, found = 384.2.

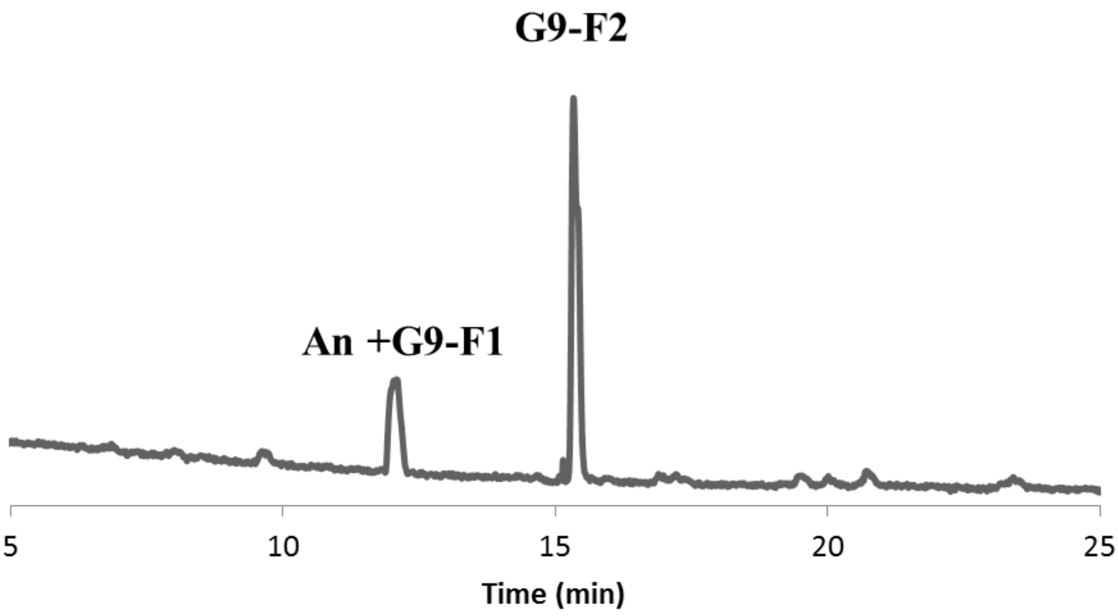

Supplementary Figure 53 | Trypsinization of G9 in the presence of 1 studied by HPLC. **G9-F1** (N-terminal) and **G9-F2** (C-terminal) are peptide fragments of **G9**. **G9-F1** and **G9-F2** were observed at 12.0 and 15.3 minutes respectively. 100 % of peptide **G9** was cut by the enzyme.

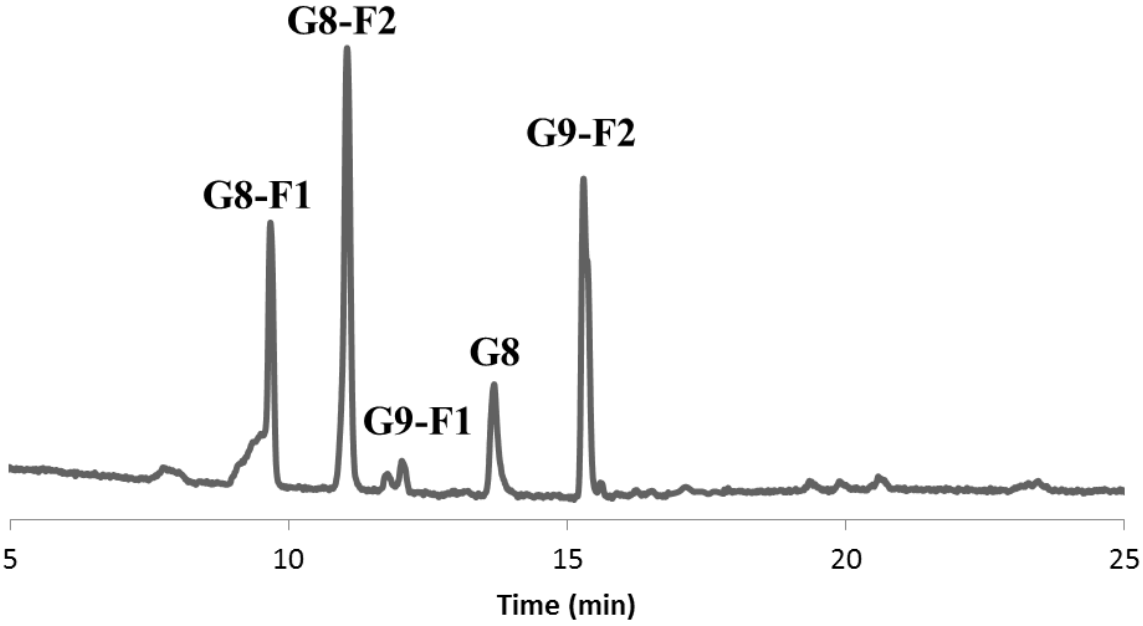

Supplementary Figure 54 | Trypsinization of a mixture of G8 and G9 studied by HPLC. 100% of peptide **G9** and 82% of peptide **G8** were cleaved by trypsin in absence of **1**. **G8–F1** (N-terminal fragment) and **G8-F2** (C-terminal fragment) were peptide fragments of **G8**. **G9-F1** (N-terminal) and **G9-F2** (C-terminal) were peptide fragments of **G9**.

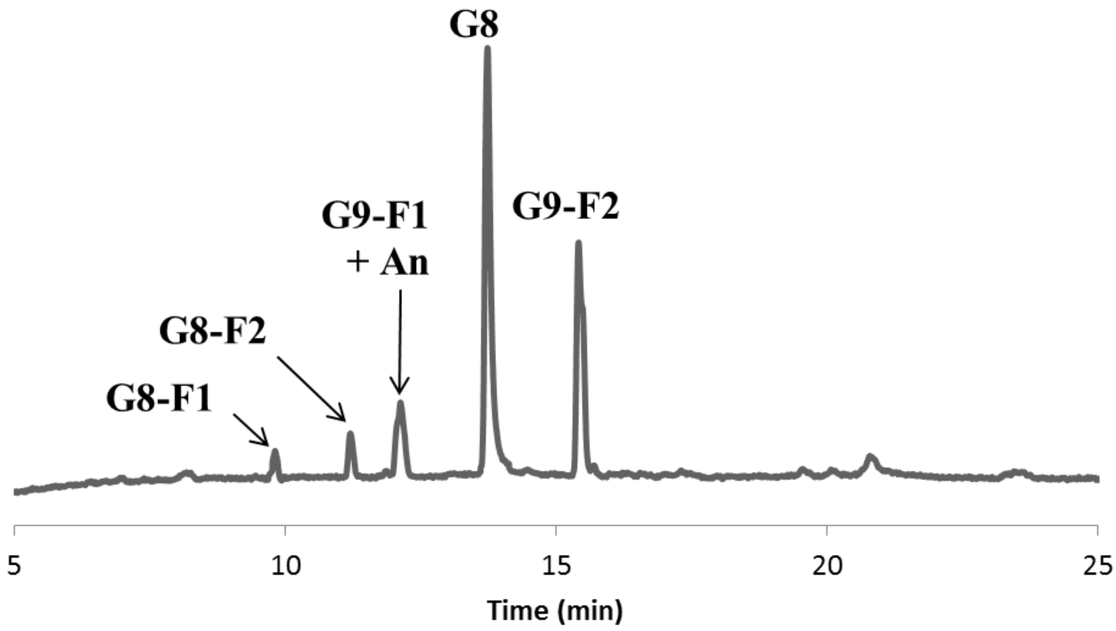

Supplementary Figure 55 | Trypsinization of a mixture of G8 and G9 in the presence of 1 studied by HPLC. 100% of peptide **G9** and only 11% of peptide **G8** were cleaved by trypsin in the presence of **1**. **G8–F1** (N-terminal fragment) and **G8-F2** (C-terminal fragment) are peptide fragments of **G8**. **G9-F1** (N-terminal) and **G9-F2** (C-terminal) are peptide fragments of **G9**. **An** is 5,6,7,8-tetrahydronaphthalen-2-amine from **1**.

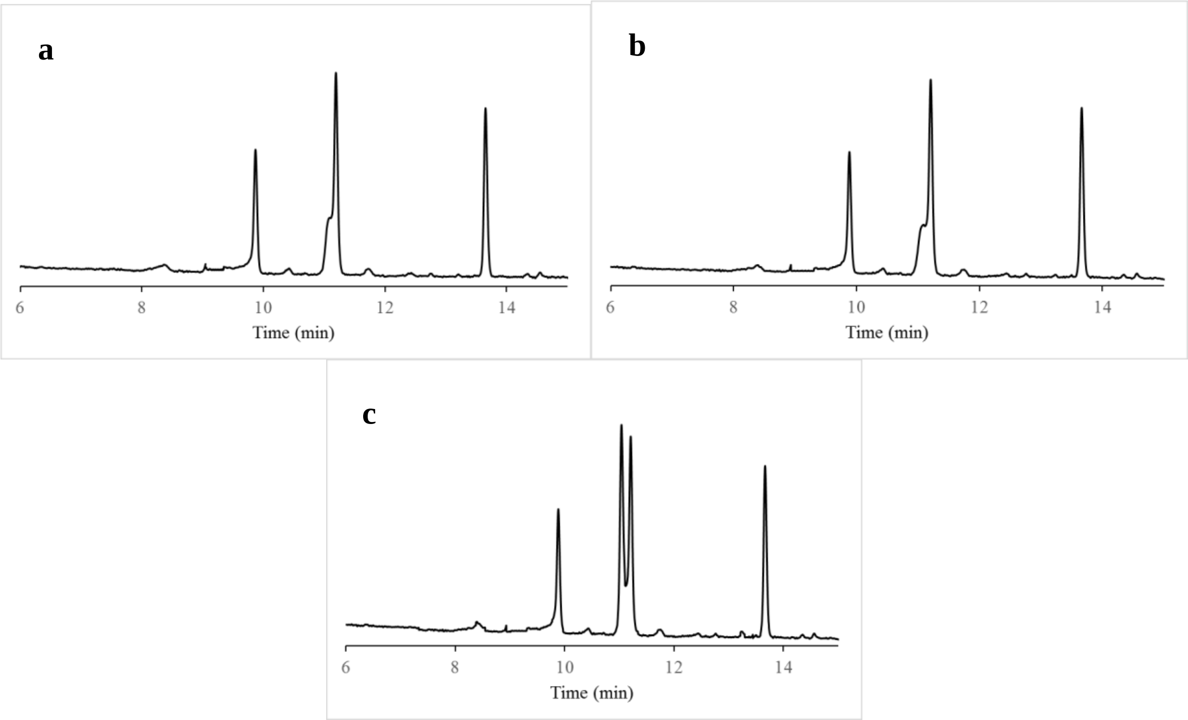

Supplementary Figure 56 | Control experiment of G8 and trypsin in the presence of free Zn-porphyrins. (**a**) Only **G8** and trypsin. (**b**) Same reaction but in the presence of Zn-porphyrin **A**. No significant difference in the peptide hydrolysis was observed when the reaction was accomplished in the presence of Zn-porphyrin **A**. However, **A** is not soluble in the mixture of solvents. (**c**) Same reaction but in presence zinc (II) meso-tetrakis-(4-sulfonatophenyl)porphyrin sodium salt. The highest peak at 11 minutes in c) is the free zinc porphyrin used in the experiment. The presence either Zn-porphyrin was not observed to have an effect upon the hydrolysis of **G8**. The same degree of hydrolysis was observed for the three cases.

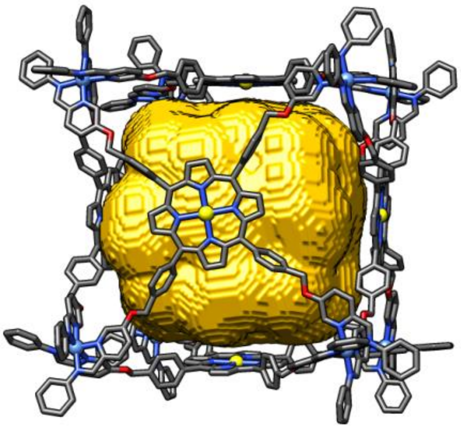

Supplementary Figure 57 | Representation of 1 cavity obtained with VOIDOO for the normal conformation. The volume of the cavity is 5600 Å^3. The average Fe^{II}···Fe^{II} distance along the cube edges are 21.8 Å. The average Zn^{II}···Zn^{II} distance between opposite porphyrins are 22.7 Å. The fused cyclic parts of the aniline residues are omitted for clarity.

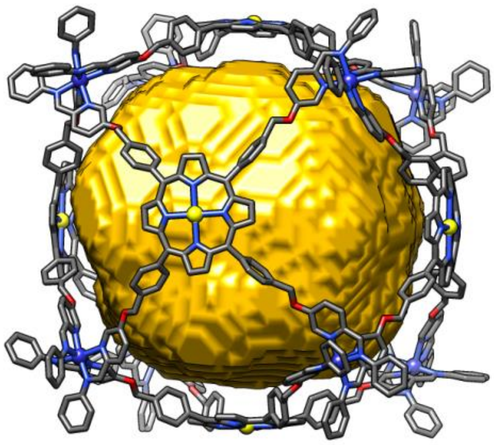

Supplementary Figure 58 | Representation of the cavity of 1 obtained with VOIDOO for the expanded conformation. The volume of the cavity is 10083 Å^3. The probe radius in this case was 6 Å. The average $Fe^{II}\cdots Fe^{II}$ distance along the cube edges are 21.0 Å. The average $Zn^{II}\cdots Zn^{II}$ distance between opposite porphyrins are 31.1 Å. The fused cyclic parts of the aniline residues are omitted for clarity.

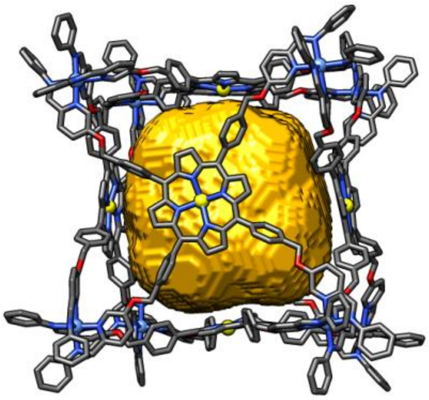

Supplementary Figure 59 | Representation of the cavity of 1 obtained with VOIDOO for the contracted conformation. The volume of the cavity is 3143 Å^3. A probe radius in this case was 4 Å. The average $Fe^{II}\cdots Fe^{II}$ distance along the cube edges are 20.1 Å. The average $Zn^{II}\cdots Zn^{II}$ distance between opposite porphyrins are 19.9 Å. The fused cyclic parts of the aniline residues are omitted for clarity.

Guest in CH_3CN	Highest average point	Lowest average point	Decrease (%)
**G1**	0.90	0.47	47.7
**G2**	0.90	0.43	52.2
**G3**	0.88	0.41	53.4
**G4**	0.87	0.39	55.2
**G5**	0.89	0.43	51.7

Supplementary Table 1 | Highest and lowest mean absorption point of 1 at 417 nm. The table above shows the percentage of signal attenuation during UV-Vis titration in CH_3CN.

Guest in 1:1 CH_3CN: H_2O	Highest average point	Lowest average point	Decrease (%)
**G1**	0.89	0.45	49.4
**G2**	0.83	0.45	45.8
**G3**	0.84	0.50	40.5
**G4**	0.85	0.46	45.9
**G5**	0.86	0.44	48.8
**G7 (300 nM)**	1.98	0.94	52.5
**G8**	0.87	0.45	48.3

Supplementary Table 2 | Highest and lowest mean absorption point of 1 at 417 nm were recorded. The table above shows the percentage of signal attenuation during UV-Vis titration in CH_3CN:H_2O (1:1).

Supplementary References

1 Roberts, D. A., Schmidt, T. W., Crossley, M. J. & Perrier, S. Tunable Self-Assembly of Triazole-Linked Porphyrin–Polymer Conjugates. *Chem. Eur. J.* **19**, 12759-12770, (2013).

2 Kuzmič, P. Program DYNAFIT for the Analysis of Enzyme Kinetic Data: Application to HIV Proteinase. *Anal. Biochem.* **237**, 260-273, (1996).

3 Satake, A. & Kobuke, Y. Dynamic supramolecular porphyrin systems. *Tetrahedron* **61**, 13-41, (2005).

4 Zarra, S., Smulders, M. M. J., Lefebvre, Q., Clegg, J. K. & Nitschke, J. R. Guanidinium Binding Modulates Guest Exchange within an [M4L6] Capsule. *Angew. Chem. Int. Ed.* **51**, 6882-6885, (2012).

5 Basak, S., Nanda, J. & Banerjee, A. Assembly of naphthalenediimide conjugated peptides: aggregation induced changes in fluorescence. *Chem. Commun.* **49**, 6891-6893, (2013).

6 CAChe WorkSystem Pro, Version 7.5.0.85 ed., Fujitsu Limited, 2000 – 2006.

7 Kleywegt, G. J. & Jones, T. A. Detection, delineation, measurement and display of cavities in macromolecular structures. *Acta Crystallogr. Sect. D* **50**, 178-185, (1994).
